# Metabolomic profiling and stable isotope labelling of *Trichomonas vaginalis* and *Tritrichomonas foetus* reveal major differences in amino acid metabolism including the production of 2-hydroxyisocaproic acid, cystathionine and S-methylcysteine

**DOI:** 10.1371/journal.pone.0189072

**Published:** 2017-12-21

**Authors:** Gareth D. Westrop, Lijie Wang, Gavin J. Blackburn, Tong Zhang, Liang Zheng, David G. Watson, Graham H. Coombs

**Affiliations:** 1 Strathclyde Institute of Pharmacy and Biomedical Science, Strathclyde University, Glasgow, United Kingdom; 2 Glasgow Polyomics, University of Glasgow, Glasgow, United Kingdom; 3 Institute of Cancer Sciences, University of Glasgow, Glasgow, United Kingdom; 4 Pediatric Translational Medicine Institute, Shanghai Children’s Medical Center, Shanghai, China; National Research Council of Italy, ITALY

## Abstract

*Trichomonas vaginalis* and *Tritrichomonas foetus* are pathogens that parasitise, respectively, human and bovine urogenital tracts causing disease. Using LC-MS, reference metabolomic profiles were obtained for both species and stable isotope labelling with D-[U-^13^C6] glucose was used to analyse central carbon metabolism. This facilitated a comparison of the metabolic pathways of *T*. *vaginalis* and *T*. *foetus*, extending earlier targeted biochemical studies. 43 metabolites, whose identities were confirmed by comparison of their retention times with authentic standards, occurred at more than 3-fold difference in peak intensity between *T*. *vaginalis* and *T*. *foetus*. 18 metabolites that were removed from or released into the medium during growth also showed more than 3-fold difference between the species. Major differences were observed in cysteine and methionine metabolism in which homocysteine, produced as a bi-product of trans-methylation, is catabolised by methionine γ-lyase in *T*. *vaginalis* but converted to cystathionine in *T*. *foetus*. Both species synthesise methylthioadenosine by an unusual mechanism, but it is not used as a substrate for methionine recycling. *T*. *vaginalis* also produces and exports high levels of S-methylcysteine, whereas only negligible levels were found in *T*. *foetus* which maintains significantly higher intracellular levels of cysteine. ^13^C-labeling confirmed that both cysteine and S-methylcysteine are synthesised by *T*. *vaginalis*; S-methylcysteine can be generated by recombinant *T*. *vaginalis* cysteine synthase using phosphoserine and methanethiol. *T*. *foetus* contained higher levels of ornithine and citrulline than *T*. *vaginalis* and exported increased levels of putrescine, suggesting greater flux through the arginine dihydrolase pathway. *T*. *vaginalis* produced and exported hydroxy acid derivatives of certain amino acids, particularly 2-hydroxyisocaproic acid derived from leucine, whereas negligible levels of these metabolites occurred in *T*. *foetus*.

## Introduction

Parabasalids are a large and diverse group of flagellated protozoa that lack mitochondria and have an anaerobic metabolism involving specialised organelles called hydrogenosomes [1]. These are adapted mitochondria that produce hydrogen as an end product of pyruvate fermentation. These protozoa have other distinctive structural features, including 3–5 flagella, a complex cytoskeleton and an enlarged golgi apparatus referred to as a parabasal body. The majority of these organisms are commensals of the enteric tract of animals but the group includes several pathogens, the most important being *Trichomonas vaginalis* and *Tritrichomonas foetus* that infect the urogenital tract of humans and cattle, respectively [2, 3].

*T*. *vaginalis* is the causative agent of trichomoniasis, an exceptionally common sexually transmitted disease of humans. Infection causes inflammation or vaginitis in about 30% of women and without treatment infection can be long lasting. In men, however, *T*. *vaginalis* infections are self-clearing and generally asymptomatic. Trichomoniasis is a cofactor in the transmission of HIV [4], the development of prostate cancer [5–7] and in pregnancy is linked to pre-term delivery and low birth weight [8, 9]. Infections can usually be controlled by 5-nitroimidazoles such as metronidazole, although drug resistance is a problem [10]. *Trichomoniasis* has been identified as a neglected parasitic infection by the CDC (http://www.cdc.gov/parasites/npi/index.html) and targeted for public health action.

*T*. *foetus* infections cause trichomoniasis in cattle where the main symptom is inflammation of the genital tract. Infection can cause sterility and in some cases involves the placenta, lungs and lymph nodes of the developing foetus and results in abortion [3]. Although primarily regarded as a bovine parasite, some strains of *T*. *foetus* can infect cats where they colonise mucosal surface of the gastro-intestinal tract and cause feline bowel disease [11]. Bovine and feline strains show a high species and organ specificity.

The *T*. *vaginalis* genome is exceptionally large and has a highly repetitive structure [12]; 65% of the genome consists of transposable elements and many protein coding genes are replicated to form high copy number gene families. A draft genome sequence of *T*. *foetus* strain K was recently published [13], making it possible to determine how closely related these trichomonads are genetically. Initial analysis revealed that 72% of ORFs in the *T*. *foetus* genome were similar to *T*. *vaginalis*, and 28% were not found in any other organism. In a separate study application of reduced representation genome sequencing revealed that gene mutations were shared between metronidazole resistant strains of *T*. *vaginalis* and *T*. *foetus* suggesting a common mechanism for development of resistance [14]. It is interesting that *T*. *foetus* has an estimated genome size of 161 Mb [13, 15] which is similarly large to the 160 Mb draft genome of *T*. *vaginalis* [12]. It also contains 62% repetitive sequences and many repeatedly duplicated genes suggesting that the genome of *T*. *foetus* evolved by massive expansion as proposed for *T*. *vaginalis* [13, 16]. Thus extreme genome size seems another common feature of *T*. *vaginalis* and *T*. *foetus* that distinguishes them from many other parabasalid species.

Similarities and differences between the metabolism of the two species, especially concerning their anaerobic energy metabolism, are known from various biochemical studies and have been reviewed extensively [17]. The major end products of glucose metabolism in *T*. *vaginalis* are glycerol, lactate and ethanol in the cytoplasm and H_2_, CO_2_ and acetate in the hydrogenosome. Alanine is produced from pyruvate as a minor end product but is not excreted. The end products formed in *T*. *foetus* are broadly similar but they do not produce lactate with instead succinate representing about 50% of the carbon released as fermentation products. Interesting differences also occur with the advent of metronidazole resistance, with metabolism shifted to lactate production in *T*. *vaginalis* but to ethanol in *T*. *foetus* [17].

A notable species difference is that *T*. *vaginalis* produces methanethiol (CH_3_SH), 2-ketobutyrate and NH_3_ by catabolism of methionine using methionine γ-lyase (MGL), although biochemical analysis failed to detect this activity in *T*. *foetus* or other trichomonad species [18]. The *T*. *vaginalis* MGL has a broad specificity and it has been proposed that H_2_S released from homocysteine by MGL is used by the cysteine synthase (CS) to produce cysteine from O-phosphoserine [19]. Cysteine is the main intracellular thiol as *T*. *vaginalis* and *T*. *foetus* do not produce glutathione or have glutathione-dependent enzymes [20].

In general, targeted biochemical analyses such as these have identified and focussed on pathways that differentiate trichomonad parasites from the mammalian host, with possible value for chemotherapeutic exploitation [21, 22] and have also highlighted differences between the species that could be linked to host range and pathogenicity. The advent of metabolomic techniques allowing non-targeted approaches for comparing the metabolomes of species has provided new opportunities for comparative analysis of the two trichomonad species. We recently compared the metabolomes of promastigotes of three *Leishmania* species and identified differences in amino acid metabolism that could, if also present in the mammalian form of the parasites, play crucial roles in determining the variations in infection [23]. We have now in this study, using the same methodology, compared the metabolites in and released by *T*. *vaginalis* and *T*. *foetus*. Samples were analysed by LCMS and metabolites were identified from their accurate mass, by comparison of their retention times with those of authentic standards, or by fragmentation analysis. Standard curves were prepared to provide an estimate of the relative concentrations. Labelling by growth of the parasites in media containing D-[U-^13^C6] glucose was used to confirm that metabolites were synthesised by the parasites and to compare the rates of production of intermediates in central carbon metabolism. In addition, a targeted analysis of *T*. *vaginalis* was carried out by comparing extracts of the parasite grown in media variations known to affect sulphur amino acid metabolism.

Our approach provides new information on the function of metabolic enzymes in *T*. *vaginalis*, an organism where methods for transfection and genetic manipulation are difficult and currently limited [14, 24].

## Materials and methods

### Trichomonad strains used in this study

The strains used in this study were *Trichomonas vaginalis* clone G3 and *Tritrichomonas foetus* clone F2. *T*. *vaginalis* G3 was the strain used for genomic sequencing [12]. *T*. *foetus* F2 is a clonal line originally derived in our laboratory from the Pfizer strain [25].

### Culture of *T*. *vaginalis* and *T*. *foetus*

*T*. *vaginalis* and *T*. *foetus* cells were maintained in 25 ml of MDM medium in sealed tubes with little gaseous phase. Cultures were initiated at a concentration of 1 x 10^5^ cells/ml and sub-cultured after 16 hours growth at 37°C when the cell concentration was 2 to 4 x 10^6^ cells/ml. The composition of MDM, which contains no added cysteine, was: 20 mg/ml Tripticase^™^ peptone (BD), 5 mg/ml maltose, 1 mg/ml ascorbic acid, 1 mg/ml KCl, 1 mg/ml KHCO_3_, 1 mg/ml KH_2_PO_4_, 0.5 mg/ml K_2_HPO_4_, 0.1 mg/ml FeSO_4_.2H_2_O, 10% (v/v) heat inactivated Horse Serum (Thermo Fisher Scientific), pH 6.2–6.3.

### Preparation of extracts for LC-MS

*T*. *vaginalis* and *T*. *foetus* were grown in the same media under identical conditions. Extracts were prepared from at least 4 independent cultures (biological replicates). For each replicate, 60 ml MDM in a 25 cm^2^ tissue culture flask was inoculated with an overnight culture to give a concentration of 10^5^ cells/ml. The lids were sealed and cultures were incubated in an upright position, with very little gaseous phase so that the culture rapidly becomes anaerobic. After growth for 16 hours at 37°C, 40 ml of the culture was transferred to a 50 ml centrifuge tube and quenched rapidly to a temperature of 4°C using a dry-ice ethanol bath, monitoring the temperature continuously with a digital thermometer.

After centrifugation at 1000 x g for 30 min at 4°C, the supernatant medium was transferred to an ice-cold 50 ml centrifuge tube and stored on ice. The cell pellet was resuspended in 40 ml ice-cold PBS and stored on ice while the cell concentration was determined using a haemocytometer. After a second centrifugation, the cells were re-suspended to give a concentration of 2 x 10^7^ cells/ml. 1 ml of the suspension was transferred to an ice-cold 1.5 ml microfuge tube and the cells were pelleted at 2000 x g for 10 min at 4°C. The pellets were mixed with 0.2 ml of extraction buffer (chloroform: methanol: water, 1:3:1) and the metabolites were extracted by shaking at 1000 rpm for 1 h at 4°C. Extracts were cleared by centrifugation at 21,000 x g for 10 min and 0.2 ml was transferred to a glass HPLC vial with a 0.2 ml insert. A blank was prepared by adding 0.2 ml of extraction buffer to an empty microfuge tube and treating in the same way as the samples. Extracts of media samples were prepared by mixing 75 μl of the sample with 0.3 ml of extraction buffer (chloroform:methanol, 1:3), shaking for 1 h at 4°C and clearing the extract by centrifugation at 21,000 x g for 10 min at 4°C. All extracts were stored at -80°C before use.

### Measurement of the protein content of cell extracts

A replicate sample containing 2 x 10^7^ cells was prepared from each culture as described above. The washed cell pellet was resuspended in 100 μl of lysis buffer (0.25 M sucrose, 0.25% (v/v) Triton X-100, 10 μM E64, 1 mM phenanthroline, 20 mM pepstatin A, 5 mM PMSF). Cells were lysed by aspiration with a pipette and the extract was cleared by centrifugation at 14,000 x g for 15 min at 4°C. The protein content was determined using the Bio-Rad protein assay (Bio-Rad).

### ^13^C labelling of *T*. *vaginalis* and *T*. *foetus* for LC-MS

MDM contains 5 mg/ml maltose as the main carbon source. In a preliminary experiment, we found that both *T*. *vaginalis* and *T*. *foetus* grew at similar rate in modified MDM without maltose supplemented with 5 mg/ml glucose. Labelling experiments were carried out using modified MDM containing 5 mg/ml D-[U-^13^C6] glucose (CK Isotopes). Cells from an overnight culture were washed by centrifugation (1000 x g for 10 mins at 4°C) and resuspension in modified MDM without glucose. 60 ml of modified MDM containing 5 mg/ml D-[U-^13^C6] glucose was inoculated with washed cells to give a concentration of 10^5^ cells/ml. Extracts for LCMS were prepared after 2 h, 6 h or 16 h growth at 37°C as described above. The medium contained no maltose and 100% of the available glucose was labelled.

### LC-MS

The LC-MS platform for metabolic profiling consisted of an Accela 600 HPLC system combined with an Exactive (Orbitrap) mass spectrometer from Thermo Fisher Scientific (Bremen, Germany). Two complementary columns, the zwitterionic ZIC-pHILLIC column (150 mm × 4.6 mm; 3.5 μm, Merck, Germany) and the reversed phase ACE C18-AR column (150 mm × 4.6 mm; 3.5 μm, Hichrom) were used. With both systems, the injection volume was 10 μl and the flow rate was 0.3 ml/min. The ZIC-pHILLIC column was eluted using a gradient of mobile phase A, 20 mM ammonium carbonate pH 9.2, and mobile phase B, acetonitrile (ACN). The concentration of A was increased from 20% to 80% over 30 min and then held at 92% for 5 mins, before equilibrating at 20%. The mobile phases for the reverse phase column were: A, 0.1% (v/v) formic acid in H_2_0; B, 0.1% (v/v) formic acid in ACN. Mobile phase A was decreased from 95% to 10% over 30 min and held at 10% for 5 min. The settings for the mass spectrometer were as described previously [23].

### Data processing

Raw data files of metabolite standard solutions were processed using ToxID 2.1 (Thermo Fisher Scientific Inc., Hemel Hempstead, UK) as described previously [23]. After visual evaluation of the extracted ion chromatograms, the retention times of the standards were used to calibrate IDEOM v19 [26]. Raw files of sample metabolites were processed as described previously [23]. Briefly, the data files were converted to mzXML open format using msConvert (ProteoWizard). Chromatograms were extracted using XCMS and stored in PeakML before aligning replicate peaks and combining them using mzMatch.R. After noise filtering and gap filling a CSV file was generated and imported into IDEOM v19 for metabolite identification based on accurate mass (± 3 ppm) and retention time prediction. The lipids and peptides were excluded from the lists of putatively identified metabolites in the biological samples.

### Analysis of ^13^C labelled metabolites

Raw data from LCMS of the ^13^C-labelled extracts was processed to generate a combined PeakML file as described above. This was further analysed using mzMatch-ISO in R [27, 28], generating a.pdf file containing chromatograms used to check peak-shape and retention time and a tab-delineated file detailing peak height for each isopotologue which was used to calculate percentage labelling.

### Metabolite identification

The identities of a subset of metabolites were confirmed by accurate mass and in addition either by matching the sample retention time to that of an authentic standard (± 0.2 min) or by obtaining MS^2^ spectra as described previously [23]. The confirmed metabolites correspond to the metabolic standards initiative (MSI) level 1. Metabolites putatively identified by accurate mass and predicted retention time correspond to MSI level 2.

### Assay for cysteine synthase and S-methylcysteine synthase activity

Recombinant *T*. *vaginalis* cysteine synthase (CS) and *T*. *vaginalis* methionine γ-lyase type 1 (MGL1) were expressed and purified as described previously [19, 29]. Specific activities using standard assays were 13 μmol/mg CS (with 3 mM sodium sulfide (Na_2_S) and 50 mM O-phosphoserine (OPS)) and 8 μmol/min/mg MGL (with 3 mM homocysteine). The activity of CS in the production of S-methylcysteine (SMC) from -OPS and sodium methanethiolate (CH_3_S.Na) was measured in a coupled assay using MGL1 and lactate dehydrogenase (LDH). The reaction was carried out in 1 ml of 200 mM potassium phosphate pH 7.8, 10 mM OPS, 3 mM CH_3_S.Na, 5 mM 2-ketoglutarate, 0.2 mM NADH, 60 μg MGL1, 25 units LDH (Sigma) and 2 μg CS. The CS activity was measured under the same conditions by substituting the CH_3_S.Na with 3 mM Na_2_S. Kinetic analysis of the SMC synthase and CS activities was carried out with a fixed (saturating) concentration of the first substrate and at least 6 different concentrations of the second. Values for *K*_m_ and V_max_ were determined by plotting the data using GraFit 5 (Erithacus Software).

### Identification of genes for metabolic enzymes in the draft genome nucleotide sequence of *T*. *foetus*

Putative genes for selected metabolic enzymes were identified by tblastn search of the whole genome shotgun contigs (wgs) database, BioProject ID 345179. Model protein sequences were obtained from TrichDB (trichdb.org) for *T*. *vaginalis* or KEGG (www.genome.jp/kegg) for other organisms.

## Results

We compared the untargeted metabolomic profiles of *T*. *vaginalis* and *T*. *foetus* to obtain insights into differences in their biochemical pathways and the levels of variances of metabolites that could be associated with differences in host range and pathogenicity. Cell extracts were first analysed by LC-MS using a ZIC-pHILLIC column for the separation step and conditions previously optimised to identify the widest possible range of polar metabolites [23]. Raw data was processed and metabolites identified using IDEOM v19 [26] calibrated with retention times obtained for 240 authentic standards. The data was screened to remove lipids and peptides which cannot be accurately identified under the conditions used. From the pHILLIC column, 86 metabolites were identified by accurate mass and confirmed by comparison of their retention times with those of authentic standards (difference ≤ 0.2 minutes). The identities of another 5 metabolites were confirmed by MS^2^ analysis. The same extracts were then analysed using a reverse phase (C18AR) column for LC-MS that provided complementary coverage, allowing confirmed identification of 19 additional metabolites including polyamines. The 110 confirmed metabolites were mapped to metabolic pathways for amino acids, carbohydrates, nucleotides, vitamins and co-factors and energy metabolism with 9 metabolites belonging to no known pathway (S1 Table). 18 of these metabolites were found only in cell extracts and 3 metabolites were specific to the media. An additional 225 metabolites from cell extracts were putatively identified on the basis of accurate mass and predicted retention time in IDEOM v19 (S2 Table). 287 metabolites were putatively identified in medium samples (S3 Table).

### Overall comparison of *T*. *vaginalis* and *T*. *foetus* cell extracts

The mean peak intensity values indicating the relative levels of metabolites in the different species were calculated from results for at least 4 biological replicates. As described previously, our method for extract preparation was optimised so that the LC-MS data did not need normalising (23). In addition, we focused mainly on large differences in mean peak intensity (> 3-fold). 43 out of 107 confirmed metabolites from cell extracts of *T*. *vaginalis* and *T*. *foetus* showed greater than 3-fold differences in peak intensity between species (Table 1). The pattern observed with the different metabolites showing either relatively high or low levels in the two species could not be the result of differences in cell volume. For example *T*. *vaginalis* has 88-fold higher levels of D-gluconic acid but 20-fold lower level of succinic acid compared with *T*.*foetus*. In addition, the protein content of *T*. *vaginalis* cell extracts, an indication of cell volume, was found to be only 1.5-fold higher than *T*. *foetus*. The protein content for 2 x 10^7^ cells was 540 ± 90 μg for *T*. *vaginalis* and 360 ± 50 μg for *T*. *foetus*. This supports our choice of a three-fold change in peak intensity as the threshold for a clear difference between the species. Differences were observed in the levels of nucleotide and amino sugars, hydroxy acid derivatives of amino acids, carboxylic acids, metabolites belonging to pathways for cysteine and methionine metabolism, arginine metabolism, lysine metabolism and in the production of thioethers not previously identified in trichomonads.

**Table 1:** Metabolites from cell extracts showing more than 3-fold difference between species.

Metabolite	Formula	*T*. *vaginalis* Mean PI	*T*. *foetus* Mean PI	Tvag/Tfoe	Pathway
**Malonate**	C3H4O4	1.15E+05	3.04E+04	3.79	Pyrimidine/ β-alanine
**2-Hydroxybutanoate**	C4H8O3	3.97E+06	5.69E+04	69.84	Propanoate
**Uracil**	C4H4N2O2	6.29E+05	2.52E+06	0.25	Pyrimidine
**Creatinine**	C4H7N3O	1.48E+06	2.91E+05	5.07	Arginine and proline
**Succinate**	C4H6O4	3.48E+06	6.41E+07	0.05	Glucose metabolism
**5-Oxoproline**	C5H7NO3	6.30E+07	1.45E+07	4.35	Arginine and proline
**L-Pipecolate**	C6H11NO2	7.63E+07	8.90E+06	8.57	Lysine
**Creatine**	C4H9N3O2	8.18E+05	1.18E+05	6.93	Arginine and Proline
**HICA**	C6H12O3	1.82E+07	2.49E+06	7.3	Leucine
**L-Ornithine**	C5H12N2O2	1.45E+05	4.26E+05	0.34	Arginine and Proline
**Malate**	C4H6O5	8.58E+06	2.08E+06	4.12	Glucose metabolism
**SMC**	C4H9NO2S	1.15E+06	5.43E+03	212.62	Unknown
**Hypoxanthine**	C5H4N4O	6.47E+06	9.57E+05	6.76	Purine
**Histidinal**	C6H9N3O	5.05E+05	4.00E+04	12.63	Histidine
**4-guanidinobutanoate**	C5H11N3O2	2.49E+05	4.96E+04	5.03	Arginine and Proline
**2-ketoglutarate**	C5H6O5	5.42E+05	3.75E+06	0.14	Glutamate
**L-Lysine**	C6H14N2O2	1.47E+06	6.02E+06	0.24	Lysine
**Imidazolone lactate**	C6H8N2O3	1.30E+06	1.38E+04	94.1	Histidine
**N6-Methyl-L-lysine**	C7H16N2O2	2.12E+04	2.72E+03	7.79	Lysine
**L-Carnitine**	C7H15NO3	3.03E+04	9.96E+03	3.04	Lysine/ acylcarnitine
**Phenyllactate**	C9H10O3	1.02E+07	9.39E+05	10.9	Phenylalanine
**N(pi)-Methyl-L-histidine**	C7H11N3O2	1.82E+05	4.50E+04	4.04	Histidine
**Indole-3-acetate**	C10H9NO2	7.57E+03	1.98E+03	3.82	Tryptophan
**L-Citrulline**	C6H13N3O3	9.40E+05	5.32E+06	0.18	Arginine
**D-Glucosamine**	C6H13NO5	5.15E+03	1.54E+03	3.34	Amino / Nucleotide sugar
**N6-Acetyl-L-lysine**	C8H16N2O3	2.62E+05	9.68E+05	0.27	Lysine
**Carboxyspermidine**	C8H19N3O2	1.00E+05	1.57E+04	6.41	Arginine and Proline
**D-Gluconic acid**	C6H12O7	1.21E+07	1.37E+05	88.35	Pentose Phosphate
**Indolepyruvate**	C11H9NO3	3.88E+03	1.13E+03	3.42	Tryptophan
**O-Acetylcarnitine**	C9H17NO4	1.11E+05	1.61E+04	6.88	Acylcarnitine
**Pantothenate**	C9H17NO5	4.51E+05	1.33E+05	3.4	Pantothenate
**L-Cystathionine**	C7H14N2O4S	1.19E+04	3.24E+05	0.04	Methionine
**L-Cystine**	C6H12N2O4S2	7.27E+03	1.01E+05	0.07	Cysteine
**Cysteine: Homocysteine disulfide**	C7H14N2O4S2	0.00E+00	1.06E+05	0	Unknown
**Thiamin**	C12H16N4OS	2.92E+05	1.64E+06	0.18	Thiamine
**Inosine**	C10H12N4O5	4.27E+05	8.26E+04	5.17	Purine
**Guanosine**	C10H13N5O5	2.67E+03	2.30E+04	0.12	Purine
**N-Acetyl-D-Glucosamine 6-phosphate**	C8H16NO9P	9.16E+04	3.06E+04	3	Amino / Nucleotide sugar
**N-Acetylneuraminate**	C11H19NO9	8.23E+03	5.49E+04	0.15	Amino / Nucleotide sugar
**Sucrose**	C12H22O11	2.27E+05	3.61E+04	6.28	Carbohydrate
**IMP**	C10H13N4O8P	3.88E+04	7.81E+03	4.97	Purine
**SAH**	C14H20N6O5S	2.78E+04	1.53E+05	0.18	Methionine
**UDP-N-acetyl-D-glucosamine**	C17H27N3O17P2	2.49E+06	6.22E+05	3.99	Amino / Nucleotide sugar
**NADP+**	C21H28N7O17P3	1.06E+05	4.83E+05	0.22	Nicotinate and Nicotinamide

The table shows mean peak intensities (PI) from LCMS analysis of *T*. *vaginalis* and *T*. *foetus* cell extracts. Numbers represent the mean of at least four extracts from independent cultures. Tvag/Tfoe indicates the ratio of the PI values. P-values ≤0.005 (Paired T-Test, assuming equal variance). Except for malonate, 4-guanidinobutanoate and indole pyruvate where P ≤ 0.05. Full details including individual standard deviation and P values are shown in S2 Table.

HICA, 2-hydroxyisocaproate; SAH, S-adenosyl-L-homocysteine; SMC, S-methylcysteine.

Comparison of cell extracts showed that the intracellular amino acid pools of the two species were generally similar, however *T*. *foetus* cell extracts contained 5-fold higher levels of lysine whereas *T*. *vaginalis* had relatively high levels of the lysine metabolites pipecolate, N6-methyl-lysine, carnitine and N-acetyl-carnitine (Table 1 and S2 Table). Significantly, our results show that *T*. *foetus* maintains up to 20-fold higher levels of cysteine, detected as cystine by LCMS following *in-situ* oxidation. Various aspects of amino acid metabolism were analysed in more detail and the data are presented in the following sub-sections.

### Overall comparison of metabolites in media samples

In order to identify metabolites taken up or exported by the parasites, peak intensity levels in complete or uninfected media samples were compared with spent media, the supernatant after cells were removed from cultures by centrifugation (Table 2, S3 Table). 16 confirmed metabolites from *T*. *vaginalis* spent media and 6 from *T*. *foetus* showed more than 3-fold difference when compared with complete media. Comparisons of these ratios revealed that for 18 metabolites there was greater than 3-fold difference in their rate of uptake or release between the species. *T*. *vaginalis* takes up higher levels of nucleosides, phosphoserine and 5-methylthioadenosine but releases hydroxy acids and SMC. Succinate is produced and exported by *T*. *foetus*. In addition, *T*. *foetus* releases, 2.8 fold higher levels of putrescine, just outside our 3-fold threshold for a clear difference between species. As expected, spent media from cultures of both species show an increase in putrescine and a decrease in spermidine levels compared with uninfected medium (Table 2). Trichomonads can synthesise putrescine but lack S-adenosylmethionine decarboxylase required for conversion to spermidine. They can obtain spermidine from the growth medium and uptake of spermine is known to be coupled to export of putrescine by an antiporter [30]. *T*. *vaginalis* is distinct from *T*. *foetus* in importing phosphoserine, purines and purine nucleosides, methylthioadenosine and nicotinamide (Table 2).

**Table 2:** Metabolites taken up or exported into media during growth of *T*. *vaginalis* and *T*. *foetus*.

Metabolite	Formula	MDMc Mean PI	*Tvag*SPT Mean PI	*Tfoe*SPT Mean PI	*Tvag*SPT/ MDMc	*Tfoe*SPT/ MDMc	*Tvag*SPT/ *Tfoe*SPT
**Putrescine**	C4H12N2	4.34E+03	7.56E+03	2.08E+04	1.74	4.79	0.36
**2-Hydroxybutanoate**	C4H8O3	1.55E+05	4.94E+06	1.64E+05	31.85	1.06	30.15
**Succinate**	C4H6O4	1.08E+07	1.10E+07	4.35E+07	1.02	4.03	0.25
**Nicotinamide**	C6H6N2O	1.24E+06	4.49E+03	1.12E+06	0.00	0.91	0.00
**L-Pipecolate**	C6H11NO2	1.77E+06	1.87E+07	2.41E+06	10.53	1.36	7.74
**HICA**	C6H12O3	2.10E+05	1.31E+07	2.87E+05	62.44	1.37	45.59
**SMC**	C4H9NO2S	6.65E+03	2.64E+05	6.21E+03	39.70	0.93	42.57
**Hypoxanthine**	C5H4N4O	7.72E+05	8.10E+06	1.01E+06	10.49	1.30	8.04
**Ethanolamine phosphate**	C2H8NO4P	7.67E+03	1.05E+04	3.71E+04	1.37	4.84	0.28
**Spermidine**	C7H19N3	2.92E+06	1.90E+05	2.66E+05	0.07	0.09	0.71
**Guanine**	C5H5N5O	1.06E+05	1.54E+04	1.29E+05	0.15	1.21	0.12
**Phenyllactate**	C9H10O3	2.59E+05	7.41E+06	5.54E+05	28.57	2.14	13.36
**O-Phosphoserine**	C3H8NO6P	3.32E+04	3.80E+03	3.55E+04	0.11	1.07	0.11
**Indolelactate**	C11H11NO3	1.96E+04	1.11E+06	1.54E+05	56.53	7.86	7.19
**Adenosine**	C10H13N5O4	1.04E+07	5.53E+04	1.04E+07	0.01	1.00	0.01
**Inosine**	C10H12N4O5	3.29E+05	9.54E+04	3.86E+05	0.29	1.17	0.25
**Guanosine**	C10H13N5O5	4.03E+05	1.54E+04	4.98E+05	0.04	1.24	0.03
**MTA**	C11H15N5O3S	1.54E+05	6.81E+02	1.36E+05	0.00	0.89	0.00
**GDP**	C10H15N5O11P2	0.00E+00	7.30E+04	1.48E+04	INF	INF	4.95

The table shows mean peak intensities for samples from complete or uninfected MDM media (MDMc), spent media (culture supernatents) from *T*. *vaginalis* cultures (TvagSPT) and spent media from *T*. *foetus* cultures (TfoeSPT). Values represent mean from at least 4 independent cultures. Ratios of spent media and complete media (TvagSPT/MDMc) and (TfoeSPT/MDMc) indicate whether a metabolite is taken up or released from cells. Differences between species are shown by the ratio of spent media (TvagSPT/TfoeSPT). P ≤ 0.005 (Paired T-Test, assuming equal variance. Except for putrescine and spermidine, P ≤ 0.05. Full details including individual standard deviation and P values are shown in S3 Table where metabolites are also categorized according to metabolic pathways.

HICA, 2-hydroxyisocaproate, MTA, 5-methylthioadenosine; SMC, S-methylcysteine.

### Amino acids are metabolised to produce 2-hydroxy acids in *T*. *vaginalis* but not in *T*. *foetus*

2-Hydroxy acids, derived from amino acids by deamination and reduction, were detected in cell extracts of both species, but with markedly higher peak intensities in *T*. *vaginalis*. To analyse the situation in greater depth, the concentrations of the most abundant hydroxy acids together with their keto acid and amino acid precursors were determined by constructing calibration curves using authentic standards, as described previously [23]. The *T*. *vaginalis* extracts contained 28 nmol/10^8^ cells of 2-hydroxyisocaproic acid (HICA), 7-fold higher than in *T*. *foetus* (Table 3). The intracellular pools of the precursors, 2-isocaproic acid and leucine were also higher in *T*. *vaginalis*. Levels of HICA in the spent medium reached 21 μM after growth of *T*. *vaginalis* for 20 h, 55-fold higher than the concentration in the original growth medium, whereas there was no significant change in the level of HICA in the *T*. *foetus* spent medium (Table 4). Utilisation of leucine by *T*. *vaginalis* did not result in a change in the concentration of leucine in the media samples which was approximately 500 μM (Table 4). Tryptophan and phenylalanine were metabolised in a similar way with concentrations of indole lactate and phenyl lactate in *T*. *vaginalis* extracts of 14.0 and 1.3 nmol/10^8^ cells, respectively (Table 3). These results show that *T*. *vaginalis* can catabolise amino acids to produce 2-hydroxy acids at a markedly higher rate than *T*. *foetus*. Certain amino acids are used preferentially by *T*. *vaginalis* with higher levels of 2-hydroxy acids obtained from leucine and phenylalanine than from tryptophan. 2-hydroxybutanoate derived from threonine, methionine or homocysteine was also detected in *T*. *vaginalis* cell extracts (Table 1) and spent media (Table 2). Relatively high peak intensities were obtained for 2-hydroxybutanoate but this metabolite was not quantified with a calibration curve.

**Table 3:** Hydroxy acid levels in cell extracts of *T*. *vaginalis* and *T*. *foetus*.

Metabolite	Formula	*T*. *vaginalis* (nmol/10^8^ cells)	*T*. *foetus* (nmol/10^8^ cells)	*Tvag / Tfoe*
**Tryptophan**	C11H12N2O2	28.4	18.0	1.57
**Indolepyruvate**	C11H9NO3	0.0	0.1	0.69
**Indolelactate**	C11H11NO3	1.3	0.5	2.77
**Indoleacetate**	C10H9NO2	1.8	0.8	2.28
**Leucine**	C6H13NO2	263.1	161.4	1.63
**2-isocaproate**	C6H10O3	0.9	0.4	2.16
**HICA**	C6H12O3	28.4	4.2	**6.84**
**Phenylalanine**	C9H11NO2	124.9	86.4	1.44
**Phenylpuruvate**	C9H8O3	0.1	0.1	1.85
**Phenyllactate**	C9H10O3	14.0	1.3	**11.03**

Concentrations of metabolites in cell extracts (nmol/10^8^ cells) were determined from peak intensity values using standard curves from at least 4 different concentrations of the authentic standard. Tvag/Tfoe indicates the ratio of the metabolite concentration. Values in bold font represent a difference between species of more than 3-fold, P < 0.001. HICA, 2-hydroxyisocaproate.

**Table 4:** Hydroxy acid levels in spent media samples of *T*. *vaginalis* and *T*. *foetus* cultures.

Metabolite	Formula	MDMc (μM)	*Tvag*SPT (μM)	*Tfoe*SPT (μM)	*Tvag*SPT/ MDMc	*Tfoe*SPT/ MDMc	*Tvag*SPT/ *Tfoe*SPT
**Tryptophan**	C11H12N2O2	64.83	63.04	62.44	0.97	0.96	1.01
**Indolepyruvate**	C11H9NO3	0.10	0.10	0.10	1.09	1.06	1.04
**Indolelactate**	C11H11NO3	0.11	0.73	0.16	**6.71**	1.50	**4.46**
**Indoleacetate**	C10H9NO2	0.88	1.12	0.89	1.28	1.02	1.26
**Leucine**	C6H13NO2	453.68	523.67	489.31	1.15	1.08	1.07
**2-isocaproate**	C6H10O3	1.70	1.90	1.92	1.12	1.13	1.04
**HICA**	C6H12O3	0.39	21.42	0.52	**55.01**	1.34	**96.42**
**Phenylalanine**	C9H11NO2	381.04	354.08	395.27	0.93	1.04	0.90
**Phenylpuruvate**	C9H8O3	0.82	0.81	0.99	0.98	1.21	0.81
**Phenyllactate**	C9H10O3	0.11	6.47	0.33	**59.92**	**3.10**	**19.34**

Concentrations of metabolites in media samples (μM) were determined from peak intensity values using standard curves from at least 4 different concentrations of the authentic standard. Ratios of spent media and complete media (TvagSPT/MDMc) and (TfoeSPT/MDMc) indicate whether a metabolite is taken up or released from cells. Differences between species are shown by the ratio of spent media (TvagSPT/TfoeSPT). Ratios in bold font represent a difference between the species of more than 3-fold, P < 0.001. HICA, 2-hydroxyisocaproate.

### Arginine metabolism

The arginine dihydrolase pathway converts arginine to ornithine via citrulline and is present in both species [31]. In addition to providing ornithine for putrescine biosynthesis, the pathway may contribute to energy metabolism [31], producing ATP by substrate level phosphorylation. *T*. *foetus* had 5-fold higher levels of citrulline than *T*. *vaginalis* and ornithine levels were increased 3-fold (Table 1). Cell extracts of the two species showed similar levels of putrescine but *T*. *foetus* exported almost 3-fold more putrescine than *T*. *vaginalis*, as indicated by comparison of spent media samples (Table 2). Other derivatives of arginine, including the organic acid 4-guanidinobutanoate and creatinine were found at increased levels (> 5-fold) in *T*. *vaginalis* (Table 1). Proline, which can be produced from arginine via ornithine, was present at similar levels in both species although increased amounts (> 4-fold) of the proline metabolite oxoproline were found in *T*. *vaginalis* (Table 1).

### Glycolysis and central carbon metabolism

**Fig 1:**
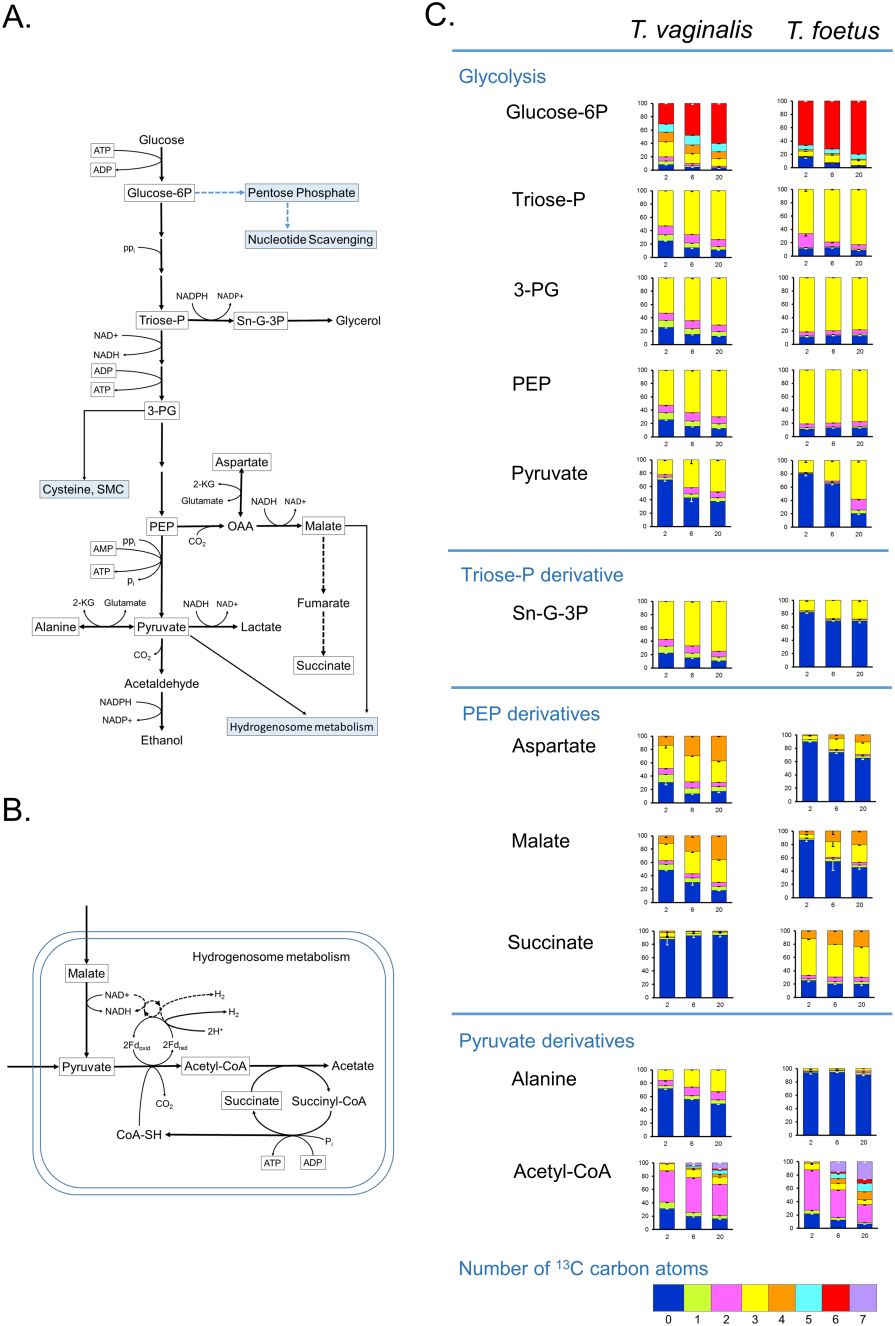
^13^C labelling of intermediates in central carbon metabolism in *T*. *vaginalis* and *T*. *foetus*. *T*. *vaginalis* and *T*. *foetus* were grown at 37°C in MDM containing 100% D-[U-^13^C6] glucose as the only carbohydrate carbon source. Cell extracts were prepared after 2 h, 6 h and 20 h in culture and analysed by LC-MS. (A) Map of glycolysis and central carbon metabolism in *T*. *vaginalis* and *T*. *foetus*. Reactions found only in *T*. *foetus* are shown by dashed lines. *T*. *vaginalis* uses pyrophosphate (ppi) dependent enzymes for the steps indicated [32]. Metabolites detected in the ^13^C labelling experiment are boxed. (B) Map of pyruvate metabolism in the hydrogenosome in *T*. *vaginalis*. Dashed lines indicate the NADH dehydrogenase complex I. Metabolites detected in the ^13^C labelling experiment are boxed. (C) ^13^C labelling of metabolites in *T*. *vaginalis* and *T*. *foetus*. Graphs show the percentage of isotopologues with different numbers of ^13^C carbon atoms.

**Fig 2:**
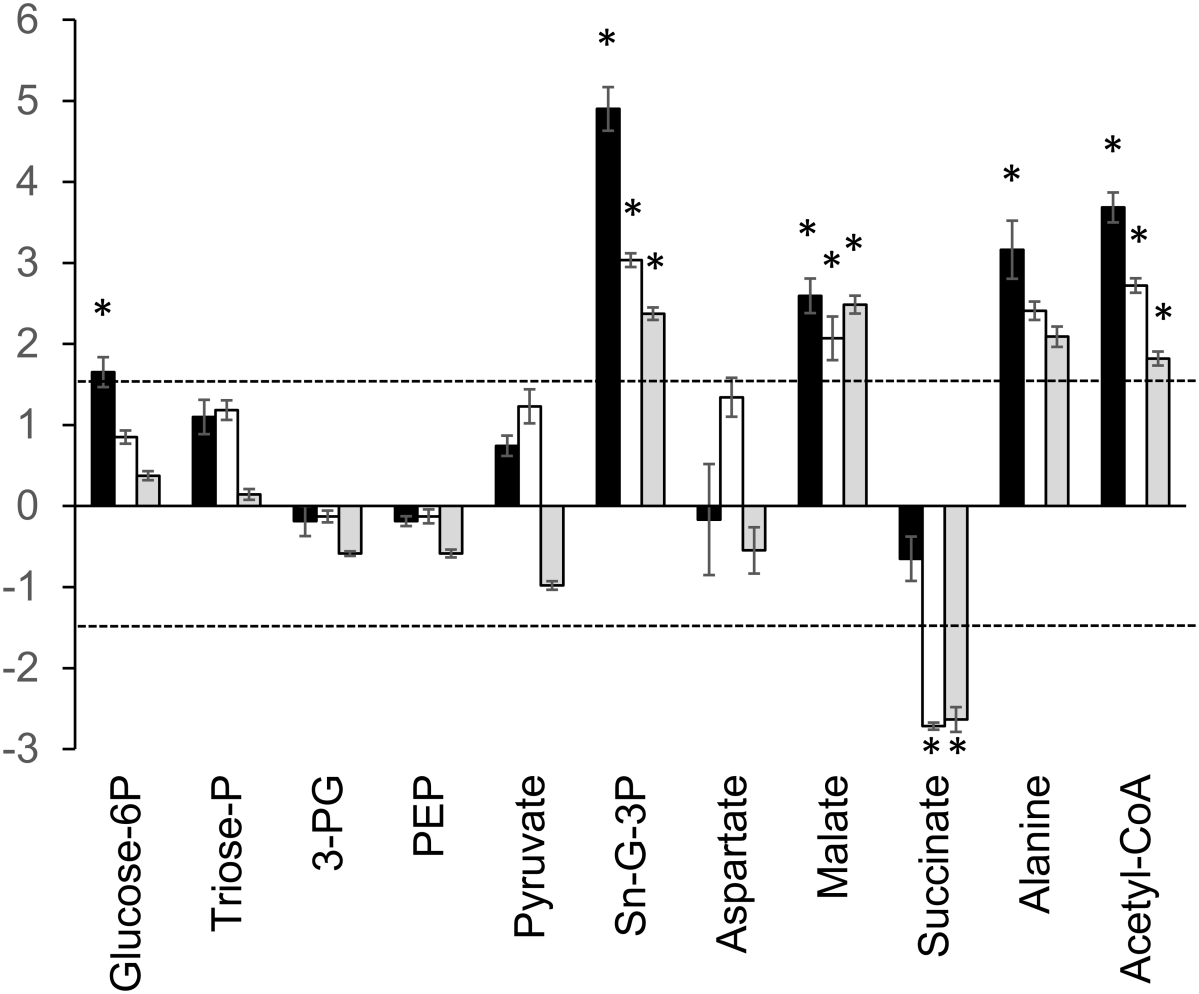
Comparison of total levels of intermediates in central carbon metabolism from ^13^C labelled extracts. Total metabolite levels represent the sum of the peak intensities of all isotopologues. Of metabolites detected in 13C-labelled extracts. Graphs show the log2 fold change in total peak intensities after 2 h, 6 h and 20 h growth. Results represent the mean of 3 independent cultures ± SD. Dashed line indicates the threshold for a definite difference between the species (3-fold or log2 1.6-fold change in metabolite level). Positive values represent a relative increase in *T*. *vaginalis*, negative values a relative increase. Glucose-6P, glucose-6-phosphate; Triose phosphate, dihydroxyacetone phosphate and glyceraldehyde-3-phosphate; 3-PG, 3-phoglycerate; PEP, phosphoenol pyruvate. * P ≤ 0.001 (Paired T-Test, assuming equal variance).

The main end products of energy metabolism in *T*. *vaginalis* are glycerol, lactate, ethanol, acetate, CO_2_ and H_2_ (Fig 1A) [17]. *T*. *foetus* differs in not producing lactate but excretes high levels of succinate. Of these metabolites, only succinate was detected by LCMS under the conditions used in this study and as expected, levels were found to be 20-fold higher in *T*. *foetus* (Table 1) as well as being excreted (Table 2). Central carbon metabolism in *T vaginalis* and *T*. *foetus* depends primarily on glycolysis because the TCA cycle is not present [17]. Fermentation of pyruvate is extended in the hydrogenosome with ATP production by substrate level phosphorylation (Fig 1B). Levels of glycolytic intermediates showed only minor differences (<2-fold) between the species (S2 Table) so we used stable isotope labelling to investigate whether the two species differ in how they utilise glucose for biosynthesis and energy production. *T*. *vaginalis* and *T*. *foetus* were grown in medium containing 100% D-[U-^13^C6] glucose and extracts were prepared after 2 h, 6 h and 20 h growth at 37°C. The change in the percentage of different ^13^C isotopologues was determined over the time course (Fig 1C) The total levels of each metabolite were calculated as the sum of the peak intensities of all isotopologues and the log2 fold change in metabolite level (*T*. *vaginalis*/*T*. *foetus*) was plotted for each time-point (Fig 2). A heat map of percentage incorporation after 20 h is also shown in the supplementary data (S1 Fig).

Even after 20 h growth, equivalent to four generations, 6-carbon labelled (6C) isotopologues of G-6P still represented only 60% of the total in *T*. *vaginalis* and 80% in *T*. *foetus* (Fig 1C and S1 Fig). Significant levels of 5C, 4C and 3C isotopologues were also detected along with unlabelled G-6P. Similarly, 3C isotopologues of pyruvate formed 50–60% of the total detected after 20 h.

^13^C labelling of glycolytic intermediates pointed to differences in glycolytic flux between the species. Although the level of incorporation into G6-P was similar, the labelling of downstream intermediates triose phosphate (LC-MS failed to resolve 3-phosphoglycerate and dihydroxy acetone phosphate), phosphoenolpyruvate (PEP) and pyruvate were a little higher in *T*. *foetus* than in *T*. *vaginalis* (Fig 1C and S1 Fig). Marked differences were seen in the labelling of metabolites of pathways branching off glycolysis. The total level of sn-glycerol-3-phosphate (sn-G-3P) in *T*. *vaginalis* at 20 h was more than 5-fold higher than in *T*. *foetus* (Table 1 and Fig 2). The rate of labelling of sn-3-GP was also markedly higher in *T*. *vaginalis* (Fig 1C and S1 Fig) confirming that it is synthesised via the triose phosphate dihydroxyacetone phosphate (DHAP).

*T*. *vaginalis* produces 4-fold higher levels of malate than *T*. *foetus* (Table 1). Both organisms showed 3 carbon and 4 carbon labelling of malate, with somewhat higher levels in *T*. *vaginalis* (Fig 1C). 3C and 4C labelled isotopologues of succinate in *T*. *foetus* represented 46% and 24% of the total at 20 h, respectively (Fig 1C and S1 Fig), whereas the relatively low levels detected in *T*. *vaginalis* were unlabelled. In *T*. *vaginalis*, malate is produced from PEP as an intermediate in energy metabolism but in *T*. *foetus*, this pathway is extended to convert malate to succinate as an end product (Fig 1A) [17]. The 3C and 4C isotopologues of aspartate would have been produced by transamination of labelled oxaloacetate. *T*. *vaginalis* showed higher rates of ^13^C labelling (Fig 1C and S1 Fig), although at 20 h the total levels of aspartate in the two species were similar (Fig 2).

Alanine is produced by transamination of pyruvate catalysed by alanine aminotransferase. 3C labelled alanine increases from 16% of the total at 2 h to 33% at 20 h in *T*. *vaginalis*, 5 fold higher than that seen in *T*. *foetus* (Fig 1C). Acetyl-CoA is produced in the hydrogenosome from pyruvate by pyruvate:ferredoxin oxidoreductase (Fig 1B). Total levels of acetyl-CoA were significantly higher in *T*. *vaginalis* (Fig 2), with 2C isotopologues making up roughly 50% of the total throughout the time course (Fig 1C). A more complex pattern of labelling was seen in *T*. *foetus* with 2C isotopologues representing 60% of the total after 2 h growth but with 2C and 7C isotopologues representing 27% and 26% of the total, respectively, after 20 h (Fig 1C). A 2C labelled acetyl group is incorporated into acetyl-CoA from 3C labelled pyruvate, but label can also be incorporated during coenzyme A biosynthesis.

### Differences in methionine metabolism

**Fig 3:**
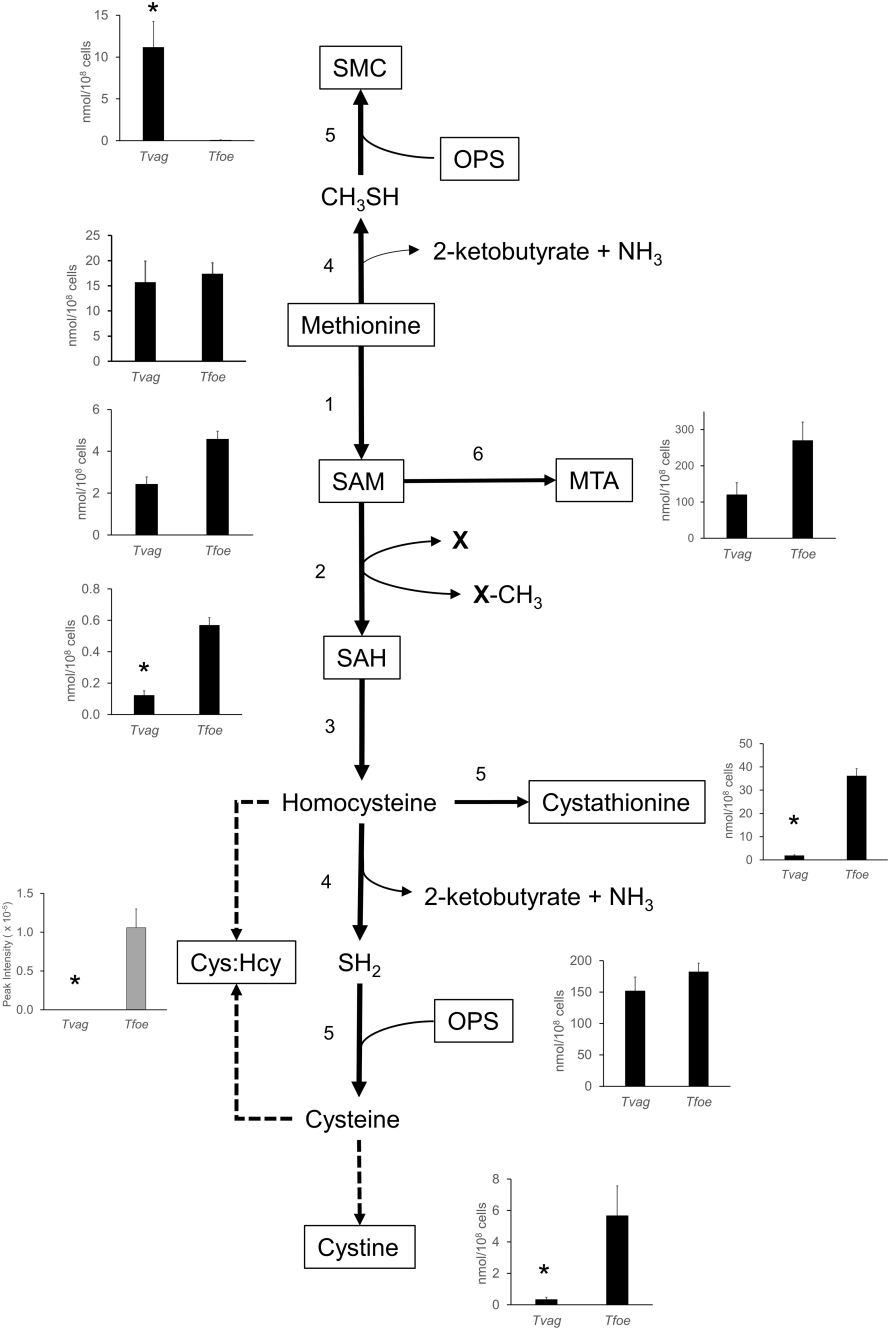
Metabolomic analysis of methionine and cysteine metabolism in *T*. *vaginalis* and *T*. *foetus*. *T*. *vaginalis* and *T*. *foetus* were grown for 20 h at 37°C in MDM which contains no added cysteine. Graphs show concentrations of metabolites (nmol/10^8^ cells) calculated from peak intensity values using standard curves. There were no standards available for the mixed disulfide of cysteine and homocysteine (Cys:Hcy disulfide) and its concentration is represented by peak intensity/10^8^ cells. Broken lines indicate the oxidation of sulfur amino acids to form disulfides. Enzymes found in the *T*. *vaginalis* genome are: 1, S-adenosylmethionine synthase; 2, S-adenosylmethionine dependent methyltransferase; 3, S-adenosylhomocysteine hydrolase; 4, methionine γ-lyase (MGL); 5, cysteine synthase (CS); 6, 1-aminocyclopropane-1-carboxylic acid (ACC) synthase. CS from *T*. *vaginalis* is a multi-functional enzyme with broad specificity, as demonstrated in later sections. * More than 3-fold difference between species. P ≤ 0.001 (Paired T-Test, assuming equal variance).

The *T*. *vaginalis* genome encodes all the enzymes required to produce homocysteine from methionine but the transsulfuration pathway for conversion of homocysteine to cysteine via cystathionine was not found (Fig 3) [12, 19]. Using calibration curves, extracts of the two species were shown to have similar levels of methionine and SAM but *T*. *foetus* contained approximately 5-fold higher levels of SAH (Fig 3). Thiols are readily oxidised in situ during LC-MS and this is most likely the reason why homocysteine was not detected in cell extracts. However, a metabolite identified as a mixed disulfide of homocysteine and cysteine by MS^2^ was found in *T*. *foetus* extracts but not in *T*. *vaginalis*, indicating that higher levels of homocysteine are produced in *T*. *foetus*. Similarly, *T*. *foetus* apparently has a higher intracellular concentration of cysteine, indicated by the detected levels of its oxidised form, the disulfide cystine. In another clear difference between the species, cystathionine was identified in *T*. *foetus* (34 nmol/10^8^ cells) but it was almost undetectable in *T*. *vaginalis* (2 nmol/10^8^ cells) (Fig 3). Both species produce relatively high levels of methylthioadenosine (MTA) with 120 nmol/10^8^ cells in *T*. *vaginalis* and 270 nmol/10^8^ cells in *T*. *foetus*. An unusual metabolite, SMC was also detected in *T*. *vaginalis* as detailed in subsequent sections.

### Labelling of intermediates in nucleotide and methionine metabolism

**Fig 4:**
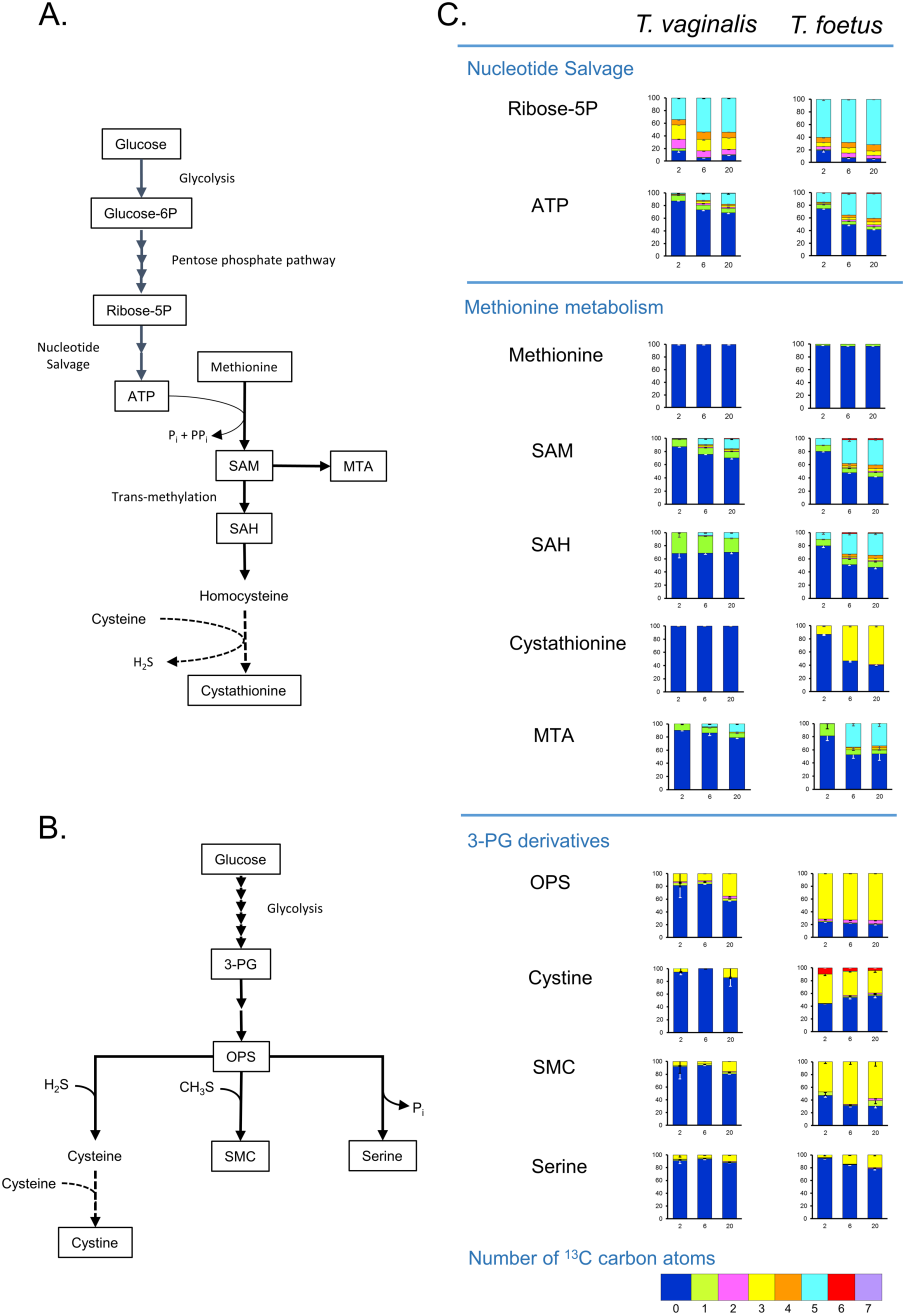
^13^C labelling of intermediates in nucleotide and methionine metabolism in *T*. *vaginalis* and *T*. *foetus*. *T*. *vaginalis* and *T*. *foetus* were grown at 37°C in MDM containing 100% D-[U-^13^C6] glucose as the only carbohydrate carbon source. Cell extracts were prepared after 2, 6 and 20 h in culture and analysed by LC-MS. (A) Map showing pathway from glucose to nucleotide and methionine metabolism in *T*. *vaginalis* and *T*. *foetus*. Reactions found only in *T*. *foetus* are shown by dashed lines. Formation of cystathionine from cysteine and homocysteine has not been confirmed. Metabolites detected in the ^13^C labelling experiment are boxed. (B) Map showing metabolites labelled with ^13^C via the glycolytic intermediate 3-phosphoglycerate. Metabolites detected in the ^13^C labelling experiment are boxed. (C) ^13^C labelling of metabolites in *T*. *vaginalis* and *T*. *foetus*. Graphs show the percentage of isotopologues with different numbers of ^13^C carbon atoms, error bars show standard deviation.

*T*. *vaginalis* and *T*. *foetus* were grown in ^13^C-glucose which was expected to label SAM via ATP (Fig 4A). The oxidative branch of the pentose phosphate pathway is active in *T*. *vaginalis* and *T*. *foetus*, with both organisms showing similar levels of ^13^C incorporation into ribose-5-phosphate after 20 h (Fig 4C, S2 Fig). Ribose-5-phosphate is used to synthesise nucleotides as shown by the presence of 5C isotopologues of UTP, GTP and ATP (Fig 4C, S2 Fig). No labelled pyrimidine or purine bases were detected indicating that nucleotides are synthesised by scavenging or recycling pathways with no evidence of *de-novo* synthesis, as reported for *T*. *vaginalis* previously [33, 34]. Higher percentages of C5 labelled nucleotides are seen in *T*. *foetus* compared to *T*. *vaginalis*, most markedly in GTP, GDP and UTP (S2 Fig). This could be explained by differences in the capacity of the two organisms for uptake unlabelled nucleosides as nucleotide precursors (Table 2).

**Fig 5:**
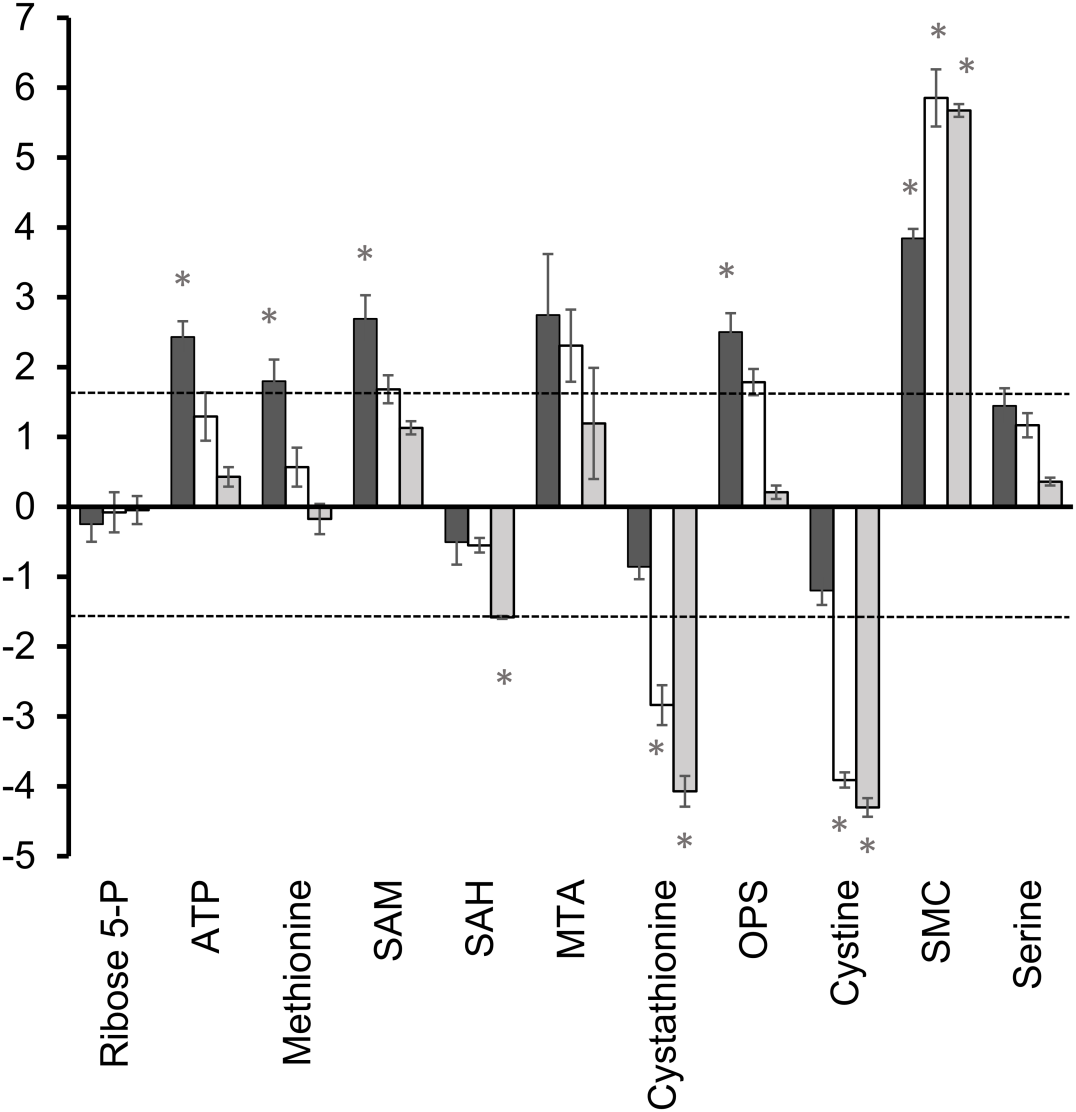
Comparison of total levels of intermediates in nucleotide and methionine metabolism from extracts of ^13^C labelled cells. Total metabolite levels represent the sum of the peak intensities of all isotopologues of metabolites detected in extracts of ^13^C labelled cells. Graphs show the log2 fold change in total peak intensities after 2, 6 and 20 h growth. Results represent the mean of 3 independent cultures ± SD. Dashed line indicates the threshold for a definite difference between the species (3-fold or log2 1.6-fold change in metabolite level). Positive values represent a relative increase in *T*. *vaginalis*, negative values a relative decrease. Ribose-5P, Ribose-5-phosphate; SAM, S-adenosylmethionine; SAH, S-adenosylhomocysteine; MTA, 5-methylthioadenosine; OPS, O-phosphoserine; SMC, S-methylcysteine. * P ≤ 0.001 (Paired T-Test, assuming equal variance).

Methionine was not labelled by ^13^C in *T*. *vaginalis* or *T*. *foetus* (Fig 4C) but 5C isotopologues of SAM and SAH were identified in both species (Fig 4C, S3 Fig). SAM is synthesised from methionine and ATP and then converted to SAH by transmethylation. The higher levels of incorporation in *T*. *foetus* are consistent with the relatively high labelling of ATP in this species (Fig 4C, S3 Fig), and do not necessarily indicate an increased rate of synthesis. C5 isotopologues of SAM and SAH were more abundant in *T*. *foetus*, again reflecting the relative incorporation of the label in the intracellular pool of ATP (Fig 4C, S3 Fig). High levels of cystathionine were detected in *T*. *foetus* and C3-labelled isotopologues increased with time, representing 60% of the total after 20 h growth (Figs 4C and 5). In contrast, the low level of cystathionine detected in *T*. *vaginalis* were not labelled (Figs 4C and 5).

### Effect of propargylglycine on methionine metabolism in *T*. *vaginalis*

**Fig 6:**
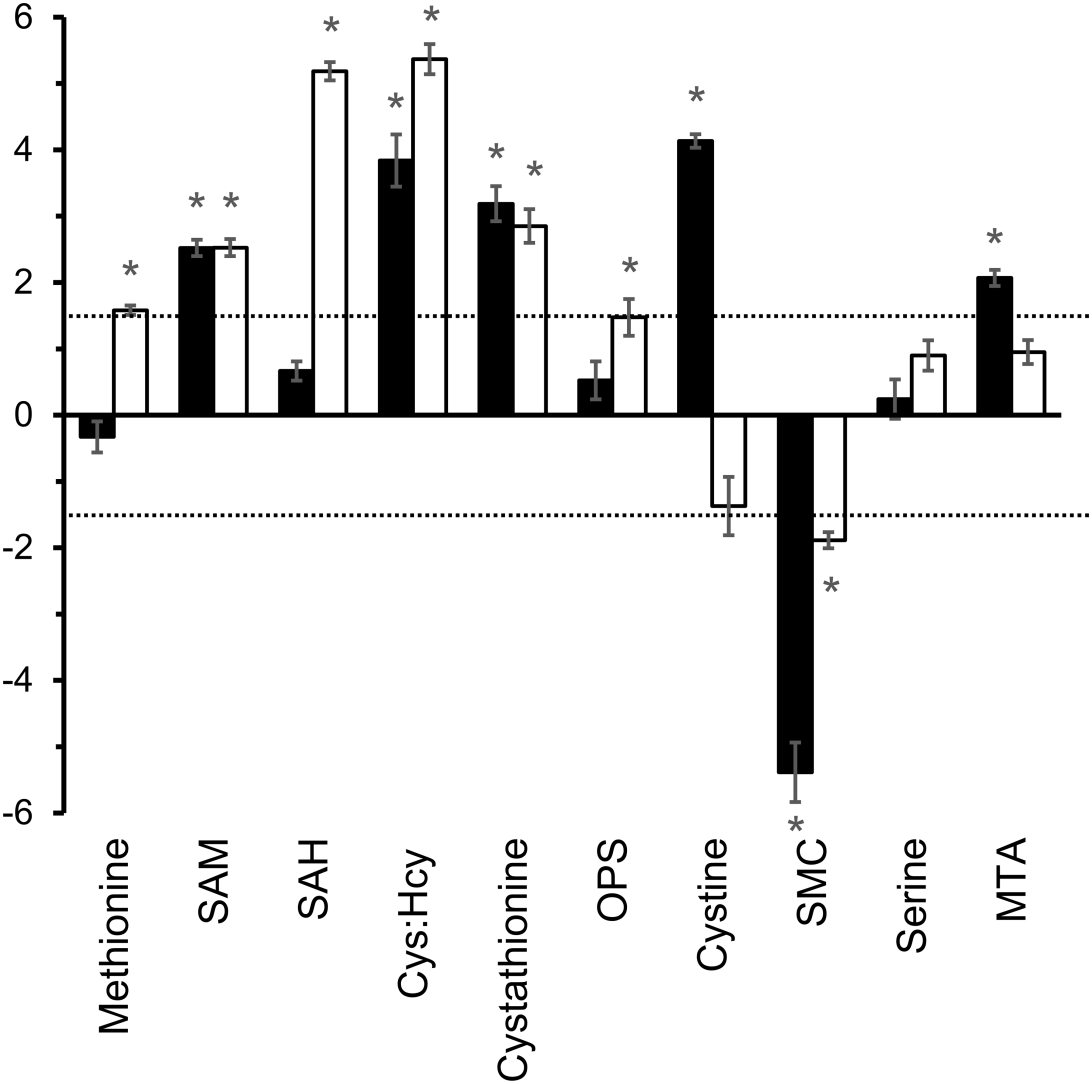
Effect of growth conditions on methionine metabolism in *T*. *vaginalis*. *T*. *vaginalis* and *T*. *foetus* were grown for 20 h at 37°C in MDM which contains no added cysteine (MDM), MDM with 10 mM cysteine (CYS) or MDM with 5 μM propargylglycine (PAG). Cell extracts were analysed by LC-MS and the ratios of metabolite peak intensities for different growth conditions were determined. The graph show the log2 of the ratios CYS/MDM and PAG/MDM ± SD. The dotted lines indicate a difference in peak intensity ≥ 3 (or log_2_ 1.6).* P ≤ 0.001 (Paired T-Test, assuming equal variance).

*T*. *vaginalis* produces MGL, a broad specificity enzyme that hydrolyses methionine, homocysteine and cysteine although biochemical analysis failed to detect this activity in cell extracts of *T*. *foetus* [18, 29, 35]. To determine whether the differences in MGL activity could account for the observed differences in methionine metabolism, *T*. *vaginalis* cultures were grown in the presence of 5 μM propargylglycine (PAG), a strong inhibitor of the *T*. *vaginalis* MGL. This resulted in a marked increase in the levels of methionine, SAM and SAH (Fig 6). The Cys:Hcy mixed disulfide was also detected in PAG-treated cell extracts, showing that more homocysteine was produced as reported previously [20]. Growth of *T*. *vaginalis* with 10 mM cysteine also affected methionine metabolism with increased levels of SAM, cystine and the Cys:Hcy mixed disulfide. (Fig 6). Interestingly, *T*. *vaginalis* has the capacity to synthesise cystathionine because when grown in medium containing added 10 mM cysteine or when cells are treated with 5 μM PAG, levels of cystathionine in cell extracts increased to 7.2 and 6.5 nmol/10^8^ cells, respectively (Fig 6).

### Identification of S-methyl-L-cysteine as a metabolite in *T*. *vaginalis* but not in *T*. *foetus*

One cellular metabolite found in the study was initially identified as homocysteine on the basis of accurate mass (m/z 135.0354). However, its retention time of 11.11 min differed from that of the authentic homocysteine standard (15.98 min). Thus, fragmentation analysis was carried out and this revealed that the metabolite was S-methyl-L-cysteine (SMC), a thioether analogue of methionine in which a methyl group is linked to the sulfur atom of cysteine (S4 Fig). The most abundant fragment (Fragment 1, 119.0161, C4H7O2S) corresponded to loss of the amino group and fragments with much lower peak intensities to loss of the carboxylate group (Fragment 2, 90.0372, C3H8NS) and to loss of the methyl-sulphur and amino groups (Fragment3, 73.0285, C3H5O2). Generation of the latter fragment arises from cleavage of a carbon-sulphur bond in SMC but requires cleavage of a high energy carbon-carbon bond in the SMC isomer homocysteine; this, makes SMC the most likely identification of the parent compound. Moreover, the fragmentation pattern was identical to those seen in MS^2^ spectra for SMC published on HMBD (http://www.hmdb.ca) and quite distinct from those obtained for homocysteine. Subsequently, we confirmed that an authentic SMC standard had the same retention time as the metabolite identified. SMC is a characteristic of *T*. *vaginalis* with only very low levels detected in *T*. *foetus* (≥ 200-fold lower peak intensity) (Table 1, Figs 3 and 5).

Using a standard curve, the concentration of SMC found in *T*. *vaginalis* extracts was 11.1 nmol/10^6^ cells but the level in *T*. *foetus* was only 0.09 nmol/10^6^ cells. SMC was also detected in spent media from *T*. *vaginalis* cultures, reaching 2.4 μM after 20 h growth, 20-fold higher than uninfected medium. These results indicate that SMC is produced in *T*. *vaginalis* and released into the medium.

### Levels of SMC in *T*. *vaginalis* are correlated with the levels of CS

*T*. *vaginalis* produces several isoforms of a cysteine synthase (CS), an enzyme linked to SMC production in *Entamoeba histolytica* [36]. To investigate whether CS could also be involved in SMC synthesis in *T*. *vaginalis* cells were grown under conditions known to affect the levels of CS expression and enzyme activity [19]. Intracellular SMC levels were found to be 50-fold lower in cells grown in medium containing 10 mM cysteine (Fig 6). Under the same conditions, CS activity was previously shown to be reduced 10-fold [19], thus raising the possibility that the *T*. *vaginalis* CS is involved in SMC synthesis. Treatment with 5 μM PAG resulted in a 4-fold reduction in the amount of SMC (Fig 6) even though cysteine synthase activity is increased [19]. However, under these conditions production of CH_3_SH, required for synthesis of SMC, is markedly reduced by inhibition of MGL.

### SMC is produced by CS *in vitro*

**Fig 7:**
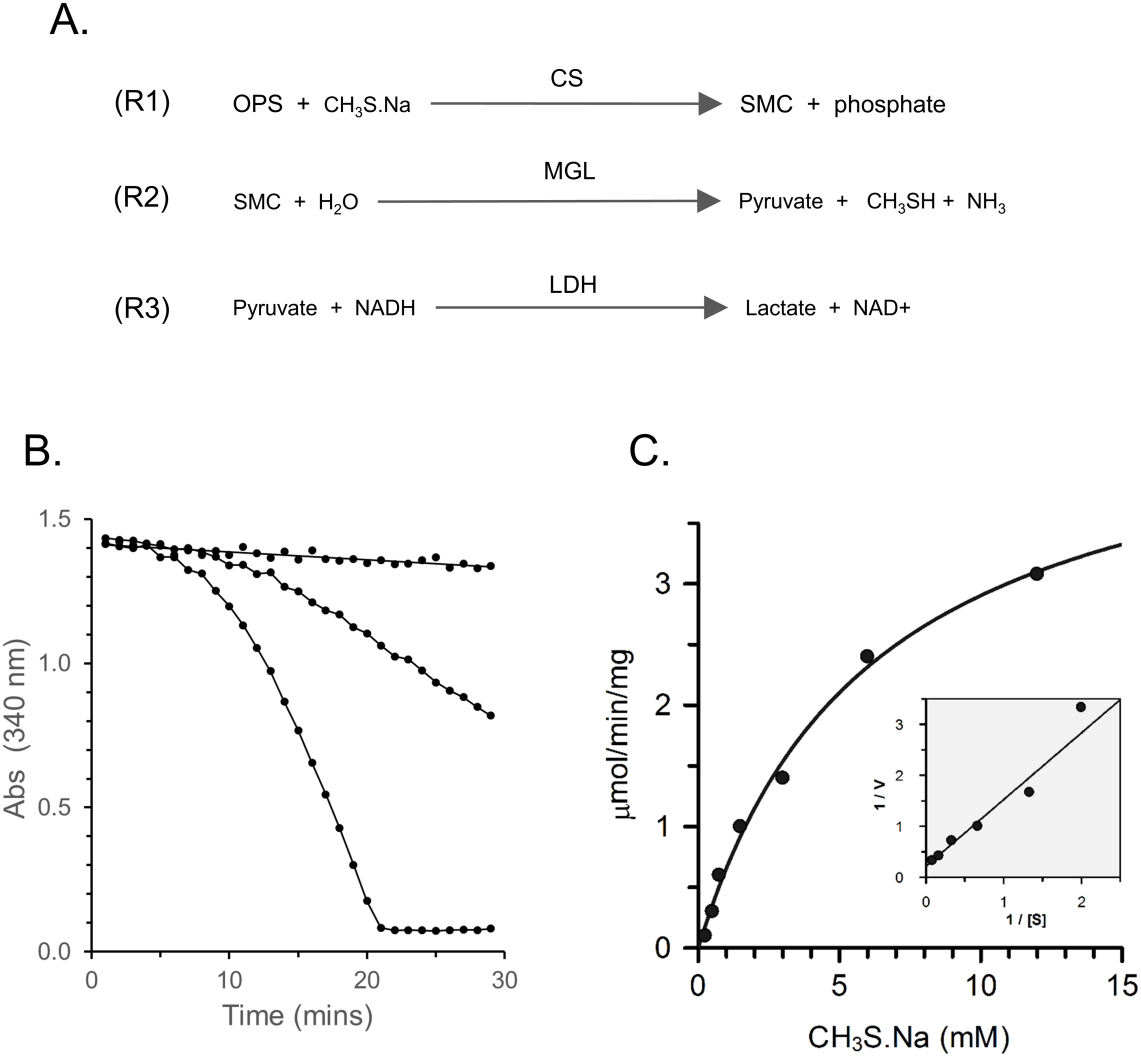
SMC biosynthesis by *T*. *vaginalis* CS. Production of SMC from OPS and CH_3_S.Na by recombinant *T*. *vaginalis* CS was determined at 37°C. (A) A coupled assay for SMC synthase. The assay couples SMC production by CS to NADH oxidation using MGL and LDH. The rate of NADH oxidation and hence the rate of SMC production can be quantified by measuring the absorbance at 340 nm. (B) The effect of increasing amounts of CS in a coupled assay for SMC biosynthesis. The reaction contained 10 mM OPS, 3 mM CH_3_SNa and 0, 1 and 5 μg/ml CS. The graph shows the rate of NADH oxidation which is equivalent to the rate of SMC biosynthesis in the coupled assay. (C) Substrate saturation curve for CH_3_SNa. Reactions contained 10 mM OPS, 85 nM CS and 0.25–12 mM sodium CH_3_SNa. A plot of enzyme activity against CH_3_SNa concentration shows Michaelis-Menten kinetics.

To determine whether the *T*. *vaginalis* CS could synthesise SMC, an *in vitro* assay was developed based on the finding that the SMC is a substrate for *T*. *vaginalis* MGL [35]. SMC was quantified in a coupled assay with recombinant *T*. *vaginalis* MGL type 1 (TvMGL1) [29] and lactate dehydrogenase (LDH). In this, SMC is catabolised by TvMGL1 to pyruvate, CH_3_SHand ammonia in the first reaction and pyruvate is reduced to lactate by LDH with oxidation of NADH in the second (Fig 7A). The production of SMC from OPS and CH_3_S.Na by CS was monitored by measuring the rate of NADH oxidation. After a lag period of about 10 min, a linear rate was maintained for more than 15 min (Fig 7B). SMC synthase activity was proportional to CS concentration in the range 25 to 125 nM. A substrate saturation curve for reactions with a fixed concentration (10 mM) of O-phosphoserine and 0.25–12 mM CH_3_S.Na exhibited Michaelis Menton kinetics with a *K*_m_ for CH_3_S.Na of 4.3 mM (Fig 7C).

Cysteine is also hydrolysed by MGL so the assay can be used to measure cysteine production by substituting Na_2_S for CH_3_SNa. This allowed a comparison of the kinetics of cysteine and SMC production by CS using the same assay (Table 5). The *K*_m_ for Na_2_S was found to be 10-fold lower than that for CH_3_SNa. The catalytic efficiency of the CS reaction with Na_2_S was 20-fold higher than for synthesis of SMC with CH_3_S.Na (Table 5).

**Table 5:** Kinetic analysis of SMC synthase and CS synthase activities of *T*. *vaginalis* CS.

*Enzyme activity*	*Substrate*	*V_max_ (nmol/min/mg)*	*K_m_ (mM)*	*k_cat_(S^-1^)*	*k_cat_/K_m_ (M^-1^s-^1^)*
***SMC synthase***	CH_3_SNa (with 10 mM OPS)	7.3 ± 2.4	4.3 ± 1.6	4.1	1.0 x 10^3^
	OPS (with 3 mM CH_3_SNa)	4.4 ± 1.4	4.6 ± 1.9	2.5	5.5 x 10^2^
***Cysteine synthase***	Na_2_S (with 50 mM OPS)	130 ± 3.6	0.4 ± 0.8	0.2	2.3 x 10^4^
	OPS (with 3 mM Na_2_S)	20.4 ± 4.2	18.2 ± 0.8	11.5	6.4 x 10^2^

### ^13^C labelling of cysteine, SMC and serine via glyceraldehyde-3-phosphate

**Fig 8:**
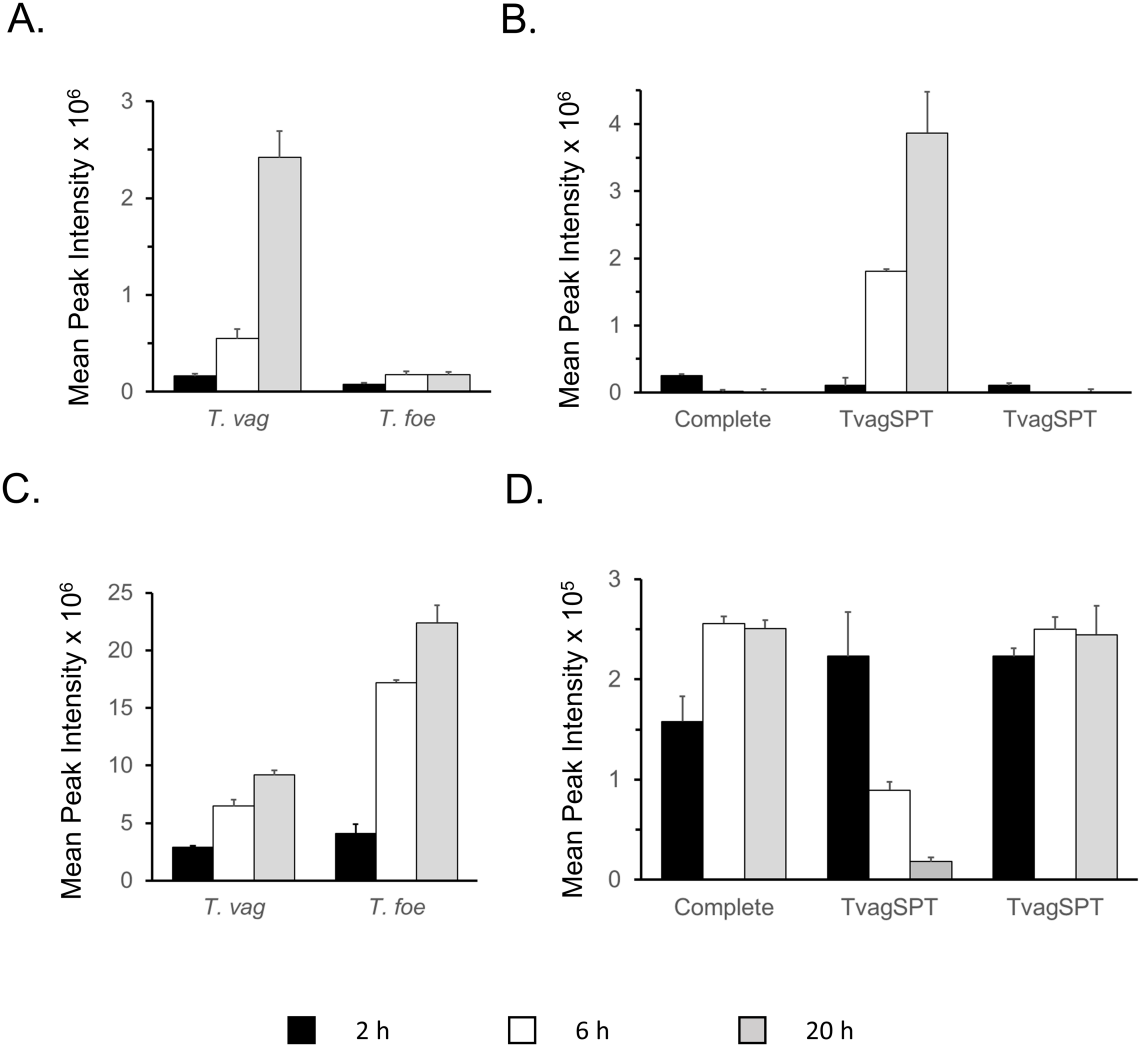
Synthesis of OPS and SMC in *T*. *vaginalis* and *T*. *foetus*. Cell extracts prepared after 2, 6 and 20 h growth at 37°C in media containing 100% D-[U-^13^C6] glucose were analysed by LC-MS. Graphs show the mean peak intensities of 3-carbon labelled isotopologues from 3 biological replicates (± SD). (A) ^13^C labelled SMC in cell extracts; (B) total SMC levels in media samples; (C) ^13^C labelled OPS in cell extracts; (D), total OPS levels in media samples. Complete, uninfected or complete media control; TvagSPT, spent media sample from *T*. *vaginalis* cultures; TfoeSPT, spent media from *T*. *foetus* cultures.

Based on measurement of cystine, *T*. *foetus* contains higher intracellular concentration of cysteine than *T*. *vaginalis* (Fig 5) and higher rates of ^13^C incorporation from glucose (Fig 4C). In contrast, ^13^C incorporation from glucose into cystine in *T*. *vaginalis* was <10% even after 20 h growth. Similarly, 80% of the SMC detected in *T*. *vaginalis* cells after 20 h growth was unlabelled, whereas the very low levels of SMC detected in *T*. *foetus* showed high percentage incorporation (Fig 4C). Clear evidence of SMC synthesis in *T*. *vaginalis* is shown, however, by the increase in peak intensity of the 3 carbon-labelled isotopologue with time (Fig 8A) and the increasing amounts of SMC released into the growth medium (Fig 8B). Intracellular levels of the ^13^C isotopologue of OPS, the precursor of cystine and SMC, increased with time (Fig 8C). However labelled OPS represented markedly lower percentage of the total in *T*. *vaginalis* (Fig 8C). Levels of OPS in the growth medium decreased 10-fold during the course of the *T*. *vaginalis* culture but there was no change in the complete medium control or in the *T*. *foetus* spent medium (Fig 8D). Overall, these results indicate that *T*. *vaginalis* imports OPS from the medium, reducing the amount that is synthesised from glucose. ^13^C labelling also revealed that both *T*. *vaginalis* and *T*. *foetus* can synthesise serine from phosphoserine, even though a search of the *T*. *vaginalis* genome database failed to detect a gene for the phosphatase required ([19]. The percentage of 3 carbon labelled serine in *T*. *foetus* was 2-fold higher than in *T*. *vaginalis*, again consistent with the lower incorporation of label into OPS in the latter.

### Identification of other thioethers including allylcysteine

The *T*. *foetus* extracts contained allycysteine, a thioether with a similar structure to SMC in which the cysteine sulphur is linked to a propenyl group rather than a methyl group. Allylcysteine was identified by comparison with an authentic standard in *T*. *foetus* extracts (S2 Table) Allylcysteine was barely detectable in *T*. *vaginalis* extracts, its sulfoxide derivative, alliin was found at 10-fold higher peak intensity than obtained for *T*. *foetus*. The mechanism of allylcysteine production is not known, although in contrast to results obtained with SMC, we detected allylcysteine in medium containing 10 mM cysteine after overnight incubation at 37°C in the absence of cells. This shows that allylcysteine can be formed spontaneously from media components under certain conditions. We conclude that allylcysteine and alliin are unlikely to be true metabolites of the trichomonads and that SMC is the only thioether detected that is synthesised by *T*. *vaginalis*.

### Genome analysis of *T*. *foetus* strain K

A draft sequence of the genome of *T*. *foetus* strain K was released while this manuscript was in preparation [13]. We therefore searched the genome for the genes of key enzymes involved in amino acid metabolism (S6 Table). *T*. *vaginalis* encodes several isoforms of a highly specific malate dehydrogenase (MDH) and a closely related lactate dehydrogenase (LDH) but *T*. *foetus* genome contained only an MDH, consistent with biochemical evidence that the parasite did not produce lactate [17]. An alignment of the *T*. *foetus* MDH proteins with the *T*. *vaginalis* MDH and LDH is shown in S5 Fig. *T*. *foetus* K encodes 3 isoforms of cysteine synthase, and the enzymes required for phosphoserine production from 3-phosphoglycerate but a gene for serine-O-acetyltransferase was not found (S6 Table). *T*. *foetus* might therefore synthesise cysteine from H_2_S and phosphoserine as reported for *T*. *vaginalis* [19]. The enzymes of the transsulfuration pathway, including CBS, were absent but unexpectedly, *T*. *foetus* appears to contain 4 isoforms of MGL (S6 Table). These *T*. *foetus* proteins have 69–72% amino acid identity with the *T*. *vaginalis* MGL isoforms and key active site residues required for PLP binding and a cysteine implicated in specificity for methionine are conserved (S6 Fig). This new finding is not consistent with previous biochemical analyses of *T*. *foetus* cell extracts [18].

## Discussion

Genomic analysis, transcriptomics and proteomics of *T*. *vaginalis* have identified potential surface proteins, secretory proteins and peptidases as virulence factors that may be involved in cell adhesion, cytotoxicity and pathogenesis [12, 37–46]. Recent studies have investigated the effect of different growth conditions on gene expression in *T*. *vaginalis* [46–48]. This is the first report of the use of metabolomics on trichomonad species, providing reference metabolic profiles of *T*. *vaginalis* and *T*. *foetus* and demonstrating how this approach can be used to identify new or unusual metabolites, compare species and identify changes in the levels of soluble metabolites occurring under different growth conditions. This study also revealed the effect of propargylglycine, an inhibitor of PLP-dependent enzymes, on the *T*. *vaginalis* metabolome.

### Central carbon metabolism in *T*. *vaginalis* and *T*. *foetus*

Glycolytic intermediates were labelled by growing cells for various times in media containing D-[U-^13^C6] glucose as the only carbohydrate carbon source. Failure to obtain 100% ^13^C labelling of G-6P suggests that trichomonads can use an alternative source of carbon skeletons for gluconeogenesis such as amino acids. This would also explain the range of different isotopologues of G-6P detected in *T*. *vaginalis*, an organism that readily takes up amino acids from the growth media [49]. These studies revealed differences in metabolic fluxes between the species. The level and rates of incorporation into G-6P and triose-P were similar, and labelling of downstream intermediates 3-PG, PEP and pyruvate were only slightly higher in *T*. *foetus* than in *T*. *vaginalis*. However, marked differences were seen in the labelling of metabolites of pathways branching off glycolysis. The rate of labelling of sn-G-3P via the triose-P DHAP was higher in *T*. *vaginalis*. Sn-G-3P is converted to glycerol, an end product of glucose metabolism in both species and it is a precursor of phosphatidic acid in glycerophospholipid biosynthesis. There were also differences in the levels of metabolites produced from PEP including malate, succinate and aspartate.

*T*. *vaginalis* encodes a PEP carboxykinase that produces OAA from PEP and CO_2_. OAA was not detected by LC-MS, but the products of its metabolism were found in extracts of both species. C3 and C4 isotopologues of malate, succinate and aspartate were identified when cells were grown in D-[U-^13^C6] glucose, with the C4 isotopologues accumulating at later times. OAA is reduced to malate by malate dehydrogenase (MDH) or transaminated to aspartate by aspartate aminotransferase (AAT). Total levels of malate were 5-fold higher in *T*. *vaginalis* and percentage ^13^C labelling was also higher, with C4 species more abundant than in *T*. *foetus*. This is no doubt due to the conversion of malate to succinate (via fumarate) which occurs in *T*. *foetus* but not in *T*. *vaginalis* [17]. *T*. *foetu*s extracts contained 20-fold higher levels of succinate and the low level of succinate detected in *T*. *vaginalis* was not significantly labelled and most likely imported from the media. These results agree with previously published data [17] and validate our methods.

The high levels of incorporation of ^13^C into aspartate, particularly in *T*. *vaginalis* extracts were not expected. In *T*. *vaginalis* multiple isoforms of AAT convert OAA and glutamate to aspartate and α-ketoglutarate [12]. However, it is not clear why *T*. *vaginalis* produces such high levels of aspartate, with 70% ^13^C labelled at 20 h, even when aspartate is available in the medium. *T*. *vaginalis* produces cytoplasmic and hydrogenosomal isoforms of MDH and AAT that could function in an aspartate:malate shuttle [40]. Antiporters required for transport of reducing equivalents across the hydrogenosomal membranes were not identified although the proteome contains several mitochondrial carrier family (MCF) proteins [40] An active aspartate:malate shuttle would explain the high rate of aspartate synthesis observed in this study. Hydrogenosomes do not have an electron transport system but NADH is required by the hydrogenosomal pyruvate:ferrodoxin oxidoreductase and reduced ferrodoxin is an electron donor for hydrogenase and the oxygen scavenging flavodiiron protein [50].

### Cysteine and SMC are produced in *T*. *vaginalis* using a bi-functional CS

Cysteine was detected predominantly in its oxidised form by LC-MS. Levels of cystine were 20-fold higher in *T*. *foetus* cell extracts than in *T*. *vaginalis* indicating that *T*. *foetus* maintains higher intracellular cysteine levels. Both organisms lack glutathione and glutathione-dependent enzymes and cysteine is thought to be a major intracellular reductant. ^13^C-labelling demonstrated that cysteine is synthesised from OPS in both trichomonad species, although the percentage incorporation was low in *T*. *vaginalis*, most likely due to its ability to take up unlabelled OPS from the medium. Surprisingly, OPS can be converted to serine in *T*. *vaginalis* and *T*. *foetus*, even though no homologues of the phosphatase required could be detected in the *T*. *vaginalis* genome [19].

We show that *T*. *vaginalis* produces SMC, an unusual metabolite that has limited distribution in nature. It is found in the seeds of leguminous plants and was recently detected in the protozoan parasite *Entamoeba histolytica* [36] which resembles *T*. *vaginalis* in lacking mitochondria and having a fermentative metabolism. *E*. *histolytica* uses CS to produce SMC, and we found that the *T*. *vaginalis* CS can also produce SMC *in vitro* from CH_3_SH and OPS. The catalytic efficiency of the *T*. *vaginalis* CS with CH_3_SH was 20-fold lower than with H_2_S in the production of cysteine whereas the *E*. *histolytica* CS showed no preference for either substrate. CH_3_SH and H_*2*_S are produced by MGL and both metabolites have been detected in *T*. *vaginalis* [51], although their intracellular concentrations are not known.

*E*. *histolytica* is dependent on an exogenous source of cysteine [36] and growth in medium containing ^13^C3, ^15^N-serine labelled SMC but not cysteine, indicating that the *E*. *histolytica* CS functioned solely as an SMC synthase [36]. In contrast, *T*. *vaginalis* grows well in medium without cysteine and levels of CS are regulated by conditions that affect cysteine availability [19]. Metabolomic profiling revealed that SMC levels are affected by the same conditions and both SMC and cysteine incorporated similar levels of ^13^C. These results suggest that the *T*. *vaginalis* CS is a bi-functional enzyme producing both cysteine and SMC. Cysteine is the main intracellular antioxidant in *T*. *foetus* whereas *T*. *vaginalis* contains both cysteine and SMC. In this study we measured an SMC concentration of 200 μM using LC-MS which compares with a concentration of 600 μM for cysteine [20]. The function of SMC is not known, but recent evidence indicates a role in other species in protection against oxidative stress [52–54] and inflammation [55].

HPLC analysis revealed that the sulfur atom of SMC is oxidised by H_2_O_2_ and the SMC-sulfoxide can be reduced by the enzyme methionine sulfoxide reductase (MSRA) *in vitro* [52]. In an animal model for Parkinson’s disease based on a recombinant *Drosophila* line expressing human α-synuclein, disease symptoms were reversed either by overexpressing human MSRA in a doubly transgenic line or by feeding with SMC, which is not produced naturally in the fruitfly [52]. It was proposed that the beneficial effects of SMC resulted from its reversible oxidation in an antioxidant system for scavenging free radicals and peroxides. In this system, consisting of SMC, MSRA, thioredoxin and thioredoxin reductase, SMC is oxidised by peroxides to form the SMC sulfoxide and this is reduced by MSRA in a thioredoxin dependent reaction. All the components of this system are found in *T*. *vaginalis* [12, 56–58] and it is possible that SMC acts as an antioxidant in *T*. *vaginalis* also.

### Role of MGL in methionine metabolism in *T*. *vaginalis*

In most organisms, including protozoan parasites, methionine is activated to SAM by methionine adenosyltransferase [59]. SAM plays a central role in cell metabolism as a methyl donor for the methylation of nucleic acids, lipids and proteins and as a source of aminopropyl groups for spermidine synthesis. SAH produced as a bi-product of SAM-dependent methyltransferase reactions is converted to homocysteine which is rapidly removed either by re-methylation to methionine or by conversion to cysteine by the transulfuration pathway. In the first, rate limiting step of transsulfuration, homocysteine is condensed with serine to form cystathionine, catalysed by cystathionine β-synthase (CBS). Cysteine is then synthesised by hydrolysis of cystathionine catalysed by cystathionine γ-lyase (CGL).

In mammals, CBS maintains low homocysteine levels, which is thought to be necessary for adequate rates of SAM-dependent methylation [60, 61]. Serum homocysteine levels are increased 10–30 fold in individuals with genetic defects in CBS or in CBS knockout mice resulting in accumulation of SAH. This is because the SAH hydrolase reaction is reversible and the kinetics *in vitro* favour the synthesis of SAH over its hydrolysis. SAH is a strong product inhibitor of many SAM-dependent methyl transferases and it has been hypothesised that increased homocysteine and SAH levels could induce epigenetic effects due to DNA hypomethylation. Tissue-specific hypomethylation has been linked to CBS knockout mutation in mice [61, 62] although conflicting results were recently reported [63].

The *T*. *vaginalis* genome encodes several isoforms of methionine adenosyl transferase and SAH hydrolase (S6 Table) [12]. Only one potential SAM-dependent methyltransferase was found in the genome although earlier, biochemical evidence demonstrated that transmethylation reactions take place in *T*. *vaginalis* and *T*. *foetus* [64]. The transulfuration pathway was not found in *T*. *vaginalis* but this species produces a broad specificity MGL that hydrolyses homocysteine, methionine and cysteine [29, 35].

In this study, the concentration of SAH in cell extracts was higher in *T*. *foetus* compared with *T*. *vaginalis*. In addition, homocysteine was detected as a mixed disulfide with cysteine in *T*. *foetus* extracts with a peak intensity of 1.0 x 10^5^ but was not detected in *T*. *vaginalis*, indicating that *T*. *foetus* cells have significantly higher levels of homocysteine. Higher levels of homocysteine and SAH would be expected to inhibit SAM-dependent methyltransferases and it is therefore interesting that lower rates of methylation have been reported in *T*. *foetus* compared with *T*. *vaginalis* [64].

When *T*. *vaginalis* was grown in media containing 5 μM propargylglycine, a potent inhibitor of MGL, the concentrations of all metabolites in the trans-methylation pathway were significantly increased, particularly SAH and homocysteine (detected as a mixed dislfide with cysteine). Methionine and homocysteine are hydrolysed by MGL so an increase in their intracellular levels was expected. SAH is a relatively poor substrate of MGL *in vitro* [35] so the > 100-fold increase in SAH we observed could be linked to the elevated intracellular levels of homocysteine and the resulting shift in the equilibrium of SAH hydrolase reaction towards synthesis of SAH. Thus it appears that one of the functions of MGL is the rapid removal of homocysteine, a function attributed to CBS in other organisms.

We show that *T*. *foetus* extracts contained high levels of cystathionine but only very low levels were detected in *T*. *vaginalis* grown under the same conditions. This suggests that *T*. *foetus* could have a different mechanism for the regulation of homocysteine levels. Cystathionine was labelled in *T*. *foetus* when cells were grown with D-[U-^13^C6] glucose and is therefore synthesised, possibly by a previously unidentified CBS activity using serine and homocysteine or by CS using cysteine and homocysteine, as reported *in vitro* for the *E*.*coli* CysM [65], an orthologue of the *T*. *vaginalis* CS [19]. Interestingly, *T*. *vaginalis* has the capacity to produce cystathionine when cells are grown in media containing added 10 mM cysteine or when MGL is inhibited by PAG, resulting in elevated intracellular homocysteine levels. We have shown previously that the *T*. *vaginalis* CS can produce *S*-2-hydroxyethyl-L-cysteine and H_2_S from cysteine and β-mercaptoethanol [19] but the production of cystathionine by the same mechanism was not investigated.

Failure to detect MGL activity in *T*. *foetus* or other trichomonad species by biochemical analysis [18] provides a simple explanation for the relatively low levels of SMC and the increased levels of homocysteine found in *T*. *foetus*. However, we found that the recently released draft genome sequence for *T*. *foetus* strain K [13] encoded 4 copies of a putative MGL. How can the previous biochemical studies and our new metabolomics analysis be reconciled with these predictions based on sequence analysis? It is possible that SMC is produced in *T*. *foetus* but is then rapidly converted to another metabolite. For example, cysteine could be formed by de-methylation of SMC. Alternatively, CH_3_SH produced from methionine could be utilised in alternative reaction. However, a more plausible explanation is that the putative MGL genes of *T*. *foetus* encode proteins adapted for another function. Like many PLP-dependent enzymes, MGL shows a range of different activities *in vitro*. Hydrolysis of methionine to produce 2-ketobutyrate, ammonia and CH_3_SH is the result of an α, γ elimination reaction and MGL enzymes from different organisms can use cysteine, homocysteine or cystathionine as alternative substrates in the same reaction with varying efficiency [66] Isoforms within a species show differences in substrate specificity as observed in *T*. *vaginalis* (29) and *E*. *hystolytica* [67] and the specificity of MGL can be altered by mutation of active site residues [29]. Cysteine and substituted serines including O-acetylserine and OPS are hydrolysed *in vitro* by an alternative α, β elimination reaction and β or γ replacement reactions are also possible. Functional analysis of the MGL from *A*. *thaliana* suggested a role in an alternative pathway for cysteine biosynthesis from methionine involving CH_3_SH [68]. The pathway was not determined but it was proposed that MGL catalysed a replacement reaction involving CH_3_SH that resulted in the release of H_2_S that could be used by CS for cysteine biosynthesis. Interestingly, kinetic analysis of the *A*. *thaliana* MGL showed that the efficiency of the α, γ -elimination reaction for hydrolysis of methionine was 3-orders of magnitude lower than that for MGLs from other organisms, leading to the suggestion that a replacement reaction is indeed preferred by the *A*. *thaliana* MGL [66]. Thus the catalysis of alternative reactions by MGL may be significant *in vivo* and MGL seems likely to perform different functions in different organisms. In this study we propose that MGL in *T*. *vaginalis* is involved in the removal of homocysteine and production of CMC via CH_3_SH. This study has shown that the same does not appear to be the case for the putative MGL identified in the *T*. *foetus* genome. Further investigation, including detailed kinetic analysis of recombinant proteins, is needed to determine the function served by the putative *T*. *foetus* MGL. We have shown that cystathionine is produced and exported by *T*. *foetus* but was detected only at very low levels in *T*. *vaginalis*. Perhaps the putative MGL isoforms of *T*. *foetus* produce cystathionine from cysteine and homocysteine by a β-replacement mechanism as reported for the *E*. *coli* CysM.

In mammals and many other organisms SAM decarboxylase produces decarboxylated SAM which acts as donor of aminopropyl groups for spermidine synthesis, producing methylthioadenosine (MTA) as a by-product. *Trichomonas* lacks SAM decarboxylase and does not synthesise spermidine, nevertheless high levels of MTA were identified in the metabolomic profiles of *T*. *vaginalis* and *T*. *foetus*. Incorporation of ^13^C confirmed that MTA was produced from SAM by an unusual mechanism. The *T*. *vaginalis* genome encodes a homolog of 1-aminocyclopropane-1-carboxylate (ACC) synthase, a plant enzyme that converts SAM to ACC and MTA [69]. As a standard, ACC gave a peak with a retention time of 12.34 but ACC was not found in cell extracts or media samples, suggesting that it is rapidly converted. In plants ACC is the precursor of the ripening hormone ethylene but there is no evidence of ethylene production in these trichomonads. MTA is a precursor in the methionine regeneration pathway in many organisms. Working with *T*. *vaginalis* cell extracts, Berger and co-workers identified an aminotransferase required for the last step in the pathway [70]. The aminotransferase converted ketomethiobutyrate to methionine *in vitro* and showed a strong preference for lysine as an amino donor. However, failure to detect ^13^C labelled methionine in our study does not support methionine recycling.

### 2-Hydroxy acids are produced in *T*. *vaginalis* using LDH

*T*. *vaginalis* converts several amino acids to 2-hydroxy acid derivatives which are exported into the medium whereas only low levels of these metabolites were detected in *T*. *foetus*. 2-hydroxy acids are produced by deamination of amino acids to form the corresponding 2-keto acid followed by reduction using an NADH dependent 2-hydroxy acid dehydrogenase. *T*. *vaginalis* encodes a branch-chain amino acid aminotransferase that transaminates leucine [71] to form 2-isocaproate. A broad specificity aspartate aminotransferase has activity with aromatic amino acids *in vitro* including phenylalanine and tryptophan [72], to produce phenylpyruvate and indole-3-pyruvate. In addition, 2-ketobutyrate can be formed from threonine by threonine dehydratase and from methionine or homocysteine by MGL. There is good evidence that these 2-keto-acid derivatives are converted to the corresponding hydroxy acids by a broad specificity LDH. Six isoforms of LDH were resolved from *T*. *vaginalis* extracts by isoelectric focussing and two of these showed high activity for ketobutyrate and 2-isocaproate [73].

One of the LDH isoforms, purified as a recombinant protein, has malate dehydrogenase (MDH) activity in addition to LDH activity [74]. This is unusual because 2-hydroxy acid dehydrogenases generally have a high specificity either for oxaloacetate or pyruvate. Based on sequence analysis it was proposed that LDH evolved from cytoplasmic MDH by gene duplication and mutation. The *T*. *vaginalis* genomic sequence encodes 10 isoforms of LDH with between 57% and 94% amino acid identity. These most likely have different substrate specificity, accounting for the wide range of hydroxy acids detected. The absence of biochemical evidence for LDH activity in *T*. *foetus* explains why only negligible amounts of hydroxy acids are detected in cell and media extracts. A search of the draft genome sequence of *T*. *foetus* strain K identified 4 isoforms of a putative MDH containing the conserved ‘specificity’ residue (S5 Fig) [74] but failed to detect any homolog of the *T*. *vaginalis* LDH (S6 Table).

Lactate is a major product of energy metabolism in *T*. *vaginalis* and the main function of LDH in *T*. *vaginalis* is likely to be redox balance. However, *T*. *vaginalis* also produces very high levels of HICA suggesting that catabolism of leucine by this route is important to the organism. HICA is produced by lactic acid bacteria and was recently shown to have antimicrobial [75–77] and anti-inflammatory activity [78]. HICA can also improve muscle recovery by inducing an increase in protein synthesis through mTOR signalling [79], which also regulates the innate immune response to bacterial, fungal or parasitic infection. The healthy vaginal microbiome is dominated by H_2_O_2_- and lactic acid-producing lactobacillus species forming a biofilm on the cervico/vaginal mucosa that protects against bacterial vaginosis, although not against trichomoniasis. *Trichomonas* infection alters the microbiome, thereby increasing vaginal pH. It is worth investigating whether HICA or other 2-hydroxy acids produced by *T*. *vaginalis* could have a role in colonisation or maintenance of *Trichomonas* infection by inhibiting the growth of competing genital bacteria or by modulating the innate immune response.

### Higher rate of arginine metabolism in *T*. *foetus*

*T*. *foetus* showed increased levels of citrulline and ornithine in cell extracts and higher levels of putrescine were found in spent media suggesting increased flux through the arginine dihydrolase pathway. Conversion of citrulline to ornithine generates carbamoyl phosphate which is further catabolised to produce ATP by substrate level phosphorylation, so arginine catabolism could increase the ATP producing capacity in the cell. This may represent a significant contribution to energy production because the rate of arginine catabolism, measured by quantifying ammonia production, was found to be comparable to the rate of carbohydrate catabolism [31]. Arginine deiminase (ADI), the first enzyme of the pathway catalysing the formation of urea and citrulline from arginine, was recently found to be located in the hydrogenosome whereas all other enzymes in the pathway are cytoplasmic [80]. The significance of the sub-cellular localisation of ADI is not known but it has been suggested that ammonium ions released by arginine catabolism could be important for maintenance of hydrogenosomal pH [80]. Alternatively, concentration of arginine in the hydrogenosome could result in increased ADI activity and higher flux. Although levels of intermediates in the arginine dihydrolase pathway were lower in *T*. *vaginalis*, other products of arginine metabolism such as 4-guanidinobutanoate were present at higher levels, suggesting that deamination or transamination of arginine may be more active in *T*. *vaginalis*.

## Conclusions

Although the metabolic profiles of *T*. *vaginalis* and *T*. *foetus* are overall very similar, this study has identified a number of polar metabolites that differ significantly in relative abundance. Our study indicates that MGL efficiently removes homocysteine *in T*. *vaginalis* and hence maintains low levels of SAH. This is important because SAH is an inhibitor of SAM-dependent methyltransferases responsible for the methylation of nucleic acids, proteins and lipids. In addition, CH_*3*_SH released from methionine by MGL is used by the *T*. *vaginalis* CS to produce SMC, and we proposed that this has a role along with cysteine as an intracellular antioxidant. CS has been investigated as a chemotherapeutic target in other organisms [81]. *T*. *vaginalis* and *T*. *foetus* are microaerophiles, they are exposed to oxygen in their natural environment and antioxidant defence mechanisms are vitally important to protect against oxidative stress. *T*. *foetus* does not produce SMC but maintains much higher intracellular levels of cysteine and appears to remove homocysteine by conversion to cystathionine. These trichomonads do not produce reactive oxygen species by aerobic respiration and the low oxygen concentrations mean that they can tolerate relatively high intracellular concentrations of cysteine [20]. The reactivity of cysteine in transition-metal autoxidation may be important in controlling oxygen levels and protecting hydrogenosomal enzymes from inactivation. *T*. *vaginalis* and *T*. *foetus* have evolved similar mechanisms for redox homeostasis in a low oxygen environment with some species-specific adaptations. It is thought that *T*. *vaginalis* acquired MGL by lateral gene transfer and previous biochemical studies failed to detect MGL activity in *T*. *foetus*. However, the recently completed draft genome sequence of *T*. *foetus* indicates that the parasite contains 4 isoforms of a putative MGL. Further studies are needed to determine the function that these putative MGL perform in *T*. *foetus*, but the differences in methionine metabolism between the species observed in this study suggest that it must differ from that of the *T*. *vaginalis* enzyme. The broad specificity *T*. *vaginalis* LDH was most likely derived from MDH by gene duplication and mutation and we confirm that LDH is not present in the *T*. *foetus* genome. In addition to producing lactate as an end product of energy metabolism, we show that *T*. vaginalis differs from *T*. *foetus* in producing markedly higher levels of the 2-hydroxy acid derivatives of certain amino acids. HICA, the hydroxy acid derived from leucine is also produced by lactic acid bacteria and has antibacterial and antibiotic properties. Early in infection, *T*. *vaginalis* must compete with the normal vaginal flora for nutrients and survive exposure to H_2_O_2_, produced by the endogenous lactobacilli. Huang and co-workers used glucose restriction to model these early conditions and observed an adaptive response that enhanced survival and increased resistance to H_2_O_2_ [47]. Under these conditions, RNAseq revealed a significant up-regulation of branch-chain aminotransferase and LDH expression [47] required for production of HICA. Although there was no increase in CS mRNA levels, increased expression of genes for OPS biosynthesis were observed. The *K*_m_ of CS for OPS is relatively high [19], thus increased phosphoserine availability could result in higher cysteine and SMC levels. Production of SMC and the release of HICA acid and other hydroxy acids by *T*. *vaginalis* could be involved in the changes in the vaginal microbiome observed during infection and may be beneficial in promoting the parasite’s survival and proliferation.

## Supporting information

S1 Table Metabolites confirmed by matching retention times with authentic standards or by MS2 analysis. (XLSX)

S2 Table All metabolites identified in cell extracts of *T*. *vaginalis* and *T*. *foetus* including confirmed metabolites and putative metabolites identified by accurate mass and predicted retention time. (XLSX)

S3 Table All metabolites identified in media samples. (XLSX)

S4 Table Concentration (μmol/10^8^ cells) of amino acids in cell extracts. (XLSX)

S5 Table Concentration (μM) of amino acids in media samples. (XLSX)

S6 Table Genes for cysteine, methionine and 2-hydroxy acid metabolism found in the draft genome of *T*. *foetus* and *T*. *vaginalis*. (XLSX)

S1 Fig ^13^C labelling of intermediates in central carbon metabolism in *T*. *vaginalis* and *T*. *foetus*. *T*. *vaginalis* and *T*. *foetus* were grown for 20 h at 37°C in MDM containing 100% D-[U-^13^C6] glucose as the only carbohydrate carbon source and cell extracts were analysed by LCMS. Heat maps of the percentage of unlabelled metabolites and isotopologues with increasing number of ^13^C atoms are shown for (A) *T*. *vaginalis* and (B) *T*. *foetus*. Numbers on the heat map refer to percentage labelling values.
(TIF)

S2 Fig ^13^C labelling of intermediates in nucleotide metabolism in *T*. *vaginalis* and *T*. *foetus*. Heat maps of the percentage of unlabelled metabolites and isotopologues with increasing number of ^13^C atoms are shown for (A) *T*. *vaginalis* and (B) *T*. *foetus*.
(TIF)

S3 Fig ^13^C labelling of intermediates in methionine metabolism in *T*. *vaginalis* and *T*. *foetus*. Heat maps of the percentage of unlabelled metabolites and their isotopologues with increasing numbers of ^13^C atoms are shown for (A) *T*. *vaginalis* and (B) *T*. *foetus*.
(TIF)

S4 Fig Identification of S-methyl-cysteine in *T*. *vaginalis* by MS^2^. A metabolite with predicted formula C4H9NO2S was analysed by MS^2^. The full length molecule was not seen in the mass spectrum. Fragments detected and their predicted formula are shown (A) in the mass spectrum and (B) in the table. The data is consistent with the structure of S-methyl-cysteine (C).
(TIF)

S5 Fig Multiple sequence alignment of putative MDH genes from *T*. *foetus*. Four putative isoforms of *T*. *foetus* MDH are aligned with MDH (TVAG_253650) and LDH (TVAG_49988). Conserved residues are in white type on a black background. Conservative amino acid differences are in white type on a grey background. The red arrow below the line indicates a residue found to be a major determinant of substrate specificity, this is arginine in *T*. *vaginalis* MDH but leucine in *T*. *vaginalis* LDH. All isoforms found in *T*. *foetus* have a positively charged residues at this position and are therefore predicted to have MDH activity. Black arrows indicate conserved residues involved in substrate binding or enzyme activity.
(TIF)

S6 FigMultiple sequence alignment of putative MGL genes from *T*. *foetus* and *T*. *vaginalis*.Sequences obtained by tblastn search of the *T*. *foetus* **and *T*. *vaginalis*** genome sequences with the *T*. *vaginalis* MGL1 (TVAG_041600) as a query sequence. Conserved residues are in white type on a black background. Conservative amino acid differences are in white type on a grey background. Symbols: ▲, active site residues involved in PLP binding; ▲, cysteine residue implicated in methionine binding.(TIF)

## References

[1] Malik SB, Brochu CD, Bilic I, Yuan J, Hess M, Logsdon JM Jr., et al Phylogeny of parasitic parabasalia and free-living relatives inferred from conventional markers vs. Rpb1, a single-copy gene. PLoS One. 2011;6(6):e20774 doi: 10.1371/journal.pone.0020774 21695260 10.1371/journal.pone.0020774 PMC3111441

[2] Leitsch D. Recent Advances in the Trichomonas vaginalis Field. F1000Res. 2016;5 26918168 10.12688/f1000research.7594.1 PMC4755396

[3] Ondrak JD. Tritrichomonas foetus Prevention and Control in Cattle. Vet Clin North Am Food Anim Pract. 2016;32(2):411–23. doi: 10.1016/j.cvfa.2016.01.010 . 27039692 10.1016/j.cvfa.2016.01.010

[4] Van Der Pol B, Kwok C, Pierre-Louis B, Rinaldi A, Salata RA, Chen PL, et al Trichomonas vaginalis infection and human immunodeficiency virus acquisition in African women. J Infect Dis. 2008;197(4):548–54. doi: 10.1086/526496 . 18275275 10.1086/526496

[5] Sutcliffe S, Giovannucci E, Alderete JF, Chang TH, Gaydos CA, Zenilman JM, et al Plasma antibodies against Trichomonas vaginalis and subsequent risk of prostate cancer. Cancer Epidemiol Biomarkers Prev. 2006;15(5):939–45. doi: 10.1158/1055-9965.EPI-05-0781 . 16702374 10.1158/1055-9965.EPI-05-0781

[6] Shui IM, Kolb S, Hanson C, Sutcliffe S, Rider JR, Stanford JL. Trichomonas vaginalis infection and risk of advanced prostate cancer. Prostate. 2016;76(7):620–3. doi: 10.1002/pros.23153 26818005 10.1002/pros.23153 PMC5002353

[7] Marous M, Huang WY, Rabkin CS, Hayes RB, Alderete JF, Rosner B, et al Trichomonas vaginalis infection and risk of prostate cancer: associations by disease aggressiveness and race/ethnicity in the PLCO Trial. Cancer Causes Control. 2017;28(8):889–98. doi: 10.1007/s10552-017-0919-6 . 28669054 10.1007/s10552-017-0919-6 PMC5554601

[8] Silver BJ, Guy RJ, Kaldor JM, Jamil MS, Rumbold AR. Trichomonas vaginalis as a cause of perinatal morbidity: a systematic review and meta-analysis. Sex Transm Dis. 2014;41(6):369–76. . 24825333 10.1097/OLQ.0000000000000134

[9] Van Der Pol B. Clinical and Laboratory Testing for Trichomonas vaginalis Infection. J Clin Microbiol. 2016;54(1):7–12. doi: 10.1128/JCM.02025-15 26491181 10.1128/JCM.02025-15 PMC4702717

[10] Leitsch D, Janssen BD, Kolarich D, Johnson PJ, Duchene M. Trichomonas vaginalis flavin reductase 1 and its role in metronidazole resistance. Mol Microbiol. 2014;91(1):198–208. doi: 10.1111/mmi.12455 24256032 10.1111/mmi.12455 PMC4437529

[11] Tolbert MK, Gookin JL. Mechanisms of Tritrichomonas foetus Pathogenicity in Cats with Insights from Venereal Trichomonosis. J Vet Intern Med. 2016;30(2):516–26. doi: 10.1111/jvim.13920 26946069 10.1111/jvim.13920 PMC4913604

[12] Carlton JM, Hirt RP, Silva JC, Delcher AL, Schatz M, Zhao Q, et al Draft genome sequence of the sexually transmitted pathogen Trichomonas vaginalis. Science. 2007;315(5809):207–12. doi: 10.1126/science.1132894 17218520 10.1126/science.1132894 PMC2080659

[13] Benchimol M, de Almeida LGP, Vasconcelos AT, de Andrade Rosa I, Reis Bogo M, Kist LW, et al Draft Genome Sequence of Tritrichomonas foetus Strain K. Genome Announc. 2017;5(16). doi: 10.1128/genomeA.00195-17 28428299 10.1128/genomeA.00195-17 PMC5399258

[14] Bradic M, Warring SD, Tooley GE, Scheid P, Secor WE, Land KM, et al Genetic indicators of drug resistance in the highly repetitive genome of Trichomonas vaginalis. Genome Biol Evol. 2017 doi: 10.1093/gbe/evx110 28633446 10.1093/gbe/evx110 PMC5522705

[15] Zubacova Z, Cimburek Z, Tachezy J. Comparative analysis of trichomonad genome sizes and karyotypes. Mol Biochem Parasitol. 2008;161(1):49–54. doi: 10.1016/j.molbiopara.2008.06.004 . 18606195 10.1016/j.molbiopara.2008.06.004

[16] Oyhenart J, Breccia JD. Evidence for repeated gene duplications in Tritrichomonas foetus supported by EST analysis and comparison with the Trichomonas vaginalis genome. Vet Parasitol. 2014;206(3–4):267–76. doi: 10.1016/j.vetpar.2014.09.024 . 25458117 10.1016/j.vetpar.2014.09.024

[17] Muller M, Mentel M, van Hellemond JJ, Henze K, Woehle C, Gould SB, et al Biochemistry and evolution of anaerobic energy metabolism in eukaryotes. Microbiol Mol Biol Rev. 2012;76(2):444–95. doi: 10.1128/MMBR.05024-11 22688819 10.1128/MMBR.05024-11 PMC3372258

[18] Thong KW, Coombs GH, Sanderson BE. L-methionine catabolism in trichomonads. Mol Biochem Parasitol. 1987;23(3):223–31. . 3496535 10.1016/0166-6851(87)90029-6

[19] Westrop GD, Goodall G, Mottram JC, Coombs GH. Cysteine biosynthesis in Trichomonas vaginalis involves cysteine synthase utilizing O-phosphoserine. J Biol Chem. 2006;281(35):25062–75. doi: 10.1074/jbc.M600688200 16735516 10.1074/jbc.M600688200 PMC2645516

[20] Westrop GD, Georg I, Coombs GH. The mercaptopyruvate sulfurtransferase of Trichomonas vaginalis links cysteine catabolism to the production of thioredoxin persulfide. J Biol Chem. 2009;284(48):33485–94. doi: 10.1074/jbc.M109.054320 19762467 10.1074/jbc.M109.054320 PMC2785193

[21] Coombs GH, Mottram JC. Trifluoromethionine, a prodrug designed against methionine gamma-lyase-containing pathogens, has efficacy in vitro and in vivo against Trichomonas vaginalis. Antimicrob Agents Chemother. 2001;45(6):1743–5. doi: 10.1128/AAC.45.6.1743-1745.2001 11353620 10.1128/AAC.45.6.1743-1745.2001 PMC90540

[22] Moya IA, Westrop GD, Coombs GH, Honek JF. Mechanistic studies on the enzymatic processing of fluorinated methionine analogues by Trichomonas vaginalis methionine gamma-lyase. Biochem J. 2011;438(3):513–21. doi: 10.1042/BJ20101986 . 21658005 10.1042/BJ20101986

[23] Westrop GD, Williams RA, Wang L, Zhang T, Watson DG, Silva AM, et al Metabolomic Analyses of Leishmania Reveal Multiple Species Differences and Large Differences in Amino Acid Metabolism. PLoS One. 2015;10(9):e0136891 doi: 10.1371/journal.pone.0136891 26368322 10.1371/journal.pone.0136891 PMC4569581

[24] Conrad MD, Bradic M, Warring SD, Gorman AW, Carlton JM. Getting trichy: tools and approaches to interrogating Trichomonas vaginalis in a post-genome world. Trends Parasitol. 2013;29(1):17–25. doi: 10.1016/j.pt.2012.10.004 23219217 10.1016/j.pt.2012.10.004 PMC3534864

[25] Mallinson DJ, Livingstone J, Appleton KM, Lees SJ, Coombs GH, North MJ. Multiple cysteine proteinases of the pathogenic protozoon Tritrichomonas foetus: identification of seven diverse and differentially expressed genes. Microbiology. 1995;141 (Pt 12):3077–85. doi: 10.1099/13500872-141-12-3077 . 8574401 10.1099/13500872-141-12-3077

[26] Creek DJ, Jankevics A, Burgess KE, Breitling R, Barrett MP. IDEOM: an Excel interface for analysis of LC-MS-based metabolomics data. Bioinformatics. 2012;28(7):1048–9. doi: 10.1093/bioinformatics/bts069 . 22308147 10.1093/bioinformatics/bts069

[27] Chokkathukalam A, Jankevics A, Creek DJ, Achcar F, Barrett MP, Breitling R. mzMatch-ISO: an R tool for the annotation and relative quantification of isotope-labelled mass spectrometry data. Bioinformatics. 2013;29(2):281–3. doi: 10.1093/bioinformatics/bts674 23162054 10.1093/bioinformatics/bts674 PMC3546800

[28] Storm J, Sethia S, Blackburn GJ, Chokkathukalam A, Watson DG, Breitling R, et al Phosphoenolpyruvate carboxylase identified as a key enzyme in erythrocytic Plasmodium falciparum carbon metabolism. PLoS Pathog. 2014;10(1):e1003876 doi: 10.1371/journal.ppat.1003876 24453970 10.1371/journal.ppat.1003876 PMC3894211

[29] McKie AE, Edlind T, Walker J, Mottram JC, Coombs GH. The primitive protozoon Trichomonas vaginalis contains two methionine gamma-lyase genes that encode members of the gamma-family of pyridoxal 5’-phosphate-dependent enzymes. J Biol Chem. 1998;273(10):5549–56. . 9488680 10.1074/jbc.273.10.5549

[30] Yarlett N, Martinez MP, Goldberg B, Kramer DL, Porter CW. Dependence of Trichomonas vaginalis upon polyamine backconversion. Microbiology. 2000;146 (Pt 10):2715–22. doi: 10.1099/00221287-146-10-2715 . 11021947 10.1099/00221287-146-10-2715

[31] Kleydman Y, Yarlett N, Gorrell TE. Production of ammonia by Tritrichomonas foetus and Trichomonas vaginalis. Microbiology. 2004;150(Pt 5):1139–45. doi: 10.1099/mic.0.26939-0 . 15133073 10.1099/mic.0.26939-0

[32] Slamovits CH, Keeling PJ. Pyruvate-phosphate dikinase of oxymonads and parabasalia and the evolution of pyrophosphate-dependent glycolysis in anaerobic eukaryotes. Eukaryot Cell. 2006;5(1):148–54. doi: 10.1128/EC.5.1.148-154.2006 16400177 10.1128/EC.5.1.148-154.2006 PMC1360263

[33] Heyworth PG, Gutteridge WE, Ginger CD. Purine metabolism in Trichomonas vaginalis. FEBS Lett. 1982;141(1):106–10. . 6282644 10.1016/0014-5793(82)80026-4

[34] Munagala NR, Wang CC. Adenosine is the primary precursor of all purine nucleotides in Trichomonas vaginalis. Mol Biochem Parasitol. 2003;127(2):143–9. . 12672523 10.1016/s0166-6851(02)00330-4

[35] Lockwood BC, Coombs GH. Purification and characterization of methionine gamma-lyase from Trichomonas vaginalis. Biochem J. 1991;279 (Pt 3):675–82. 1953661 10.1042/bj2790675 PMC1151498

[36] Husain A, Sato D, Jeelani G, Mi-ichi F, Ali V, Suematsu M, et al Metabolome analysis revealed increase in S-methylcysteine and phosphatidylisopropanolamine synthesis upon L-cysteine deprivation in the anaerobic protozoan parasite Entamoeba histolytica. J Biol Chem. 2010;285(50):39160–70. doi: 10.1074/jbc.M110.167304 20923776 10.1074/jbc.M110.167304 PMC2998131

[37] Hirt RP. Trichomonas vaginalis virulence factors: an integrative overview. Sex Transm Infect. 2013;89(6):439–43. doi: 10.1136/sextrans-2013-051105 23694938 10.1136/sextrans-2013-051105 PMC3749517

[38] Hirt RP, de Miguel N, Nakjang S, Dessi D, Liu YC, Diaz N, et al Trichomonas vaginalis pathobiology new insights from the genome sequence. Adv Parasitol. 2011;77:87–140. doi: 10.1016/B978-0-12-391429-3.00006-X . 22137583 10.1016/B978-0-12-391429-3.00006-X

[39] Yeh YM, Huang KY, Richie Gan RC, Huang HD, Wang TC, Tang P. Phosphoproteome profiling of the sexually transmitted pathogen Trichomonas vaginalis. J Microbiol Immunol Infect. 2013;46(5):366–73. doi: 10.1016/j.jmii.2012.07.010 . 22921107 10.1016/j.jmii.2012.07.010

[40] Schneider RE, Brown MT, Shiflett AM, Dyall SD, Hayes RD, Xie Y, et al The Trichomonas vaginalis hydrogenosome proteome is highly reduced relative to mitochondria, yet complex compared with mitosomes. Int J Parasitol. 2011;41(13–14):1421–34. doi: 10.1016/j.ijpara.2011.10.001 22079833 10.1016/j.ijpara.2011.10.001 PMC4437511

[41] Huang KY, Huang PJ, Ku FM, Lin R, Alderete JF, Tang P. Comparative transcriptomic and proteomic analyses of Trichomonas vaginalis following adherence to fibronectin. Infect Immun. 2012;80(11):3900–11. doi: 10.1128/IAI.00611-12 22927047 10.1128/IAI.00611-12 PMC3486053

[42] Huang KY, Chien KY, Lin YC, Hsu WM, Fong IK, Huang PJ, et al A proteome reference map of Trichomonas vaginalis. Parasitology research. 2009;104(4):927–33. doi: 10.1007/s00436-008-1274-z . 19066965 10.1007/s00436-008-1274-z

[43] de Miguel N, Lustig G, Twu O, Chattopadhyay A, Wohlschlegel JA, Johnson PJ. Proteome analysis of the surface of Trichomonas vaginalis reveals novel proteins and strain-dependent differential expression. Mol Cell Proteomics. 2010;9(7):1554–66. doi: 10.1074/mcp.M000022-MCP201 20467041 10.1074/mcp.M000022-MCP201 PMC2938091

[44] Beltran NC, Horvathova L, Jedelsky PL, Sedinova M, Rada P, Marcincikova M, et al Iron-induced changes in the proteome of Trichomonas vaginalis hydrogenosomes. PLoS One. 2013;8(5):e65148 doi: 10.1371/journal.pone.0065148 23741475 10.1371/journal.pone.0065148 PMC3669245

[45] Horvathova L, Safarikova L, Basler M, Hrdy I, Campo NB, Shin JW, et al Transcriptomic identification of iron-regulated and iron-independent gene copies within the heavily duplicated Trichomonas vaginalis genome. Genome Biol Evol. 2012;4(10):1017–29. doi: 10.1093/gbe/evs078 22975721 10.1093/gbe/evs078 PMC3490414

[46] Gould SB, Woehle C, Kusdian G, Landan G, Tachezy J, Zimorski V, et al Deep sequencing of Trichomonas vaginalis during the early infection of vaginal epithelial cells and amoeboid transition. Int J Parasitol. 2013;43(9):707–19. doi: 10.1016/j.ijpara.2013.04.002 . 23688716 10.1016/j.ijpara.2013.04.002

[47] Huang KY, Chen YY, Fang YK, Cheng WH, Cheng CC, Chen YC, et al Adaptive responses to glucose restriction enhance cell survival, antioxidant capability, and autophagy of the protozoan parasite Trichomonas vaginalis. Biochim Biophys Acta. 2014;1840(1):53–64. doi: 10.1016/j.bbagen.2013.08.008 . 23958562 10.1016/j.bbagen.2013.08.008

[48] Fang YK, Huang KY, Huang PJ, Lin R, Chao M, Tang P. Gene-expression analysis of cold-stress response in the sexually transmitted protist Trichomonas vaginalis. J Microbiol Immunol Infect. 2015;48(6):662–75. doi: 10.1016/j.jmii.2014.07.013 . 25440978 10.1016/j.jmii.2014.07.013

[49] Zuo X, Lockwood BC, Coombs GH. Uptake of amino acids by the parasitic, flagellated protist Trichomonas vaginalis. Microbiology. 1995;141 (Pt 10):2637–42. doi: 10.1099/13500872-141-10-2637 . 7582024 10.1099/13500872-141-10-2637

[50] Smutna T, Goncalves VL, Saraiva LM, Tachezy J, Teixeira M, Hrdy I. Flavodiiron protein from Trichomonas vaginalis hydrogenosomes: the terminal oxygen reductase. Eukaryot Cell. 2009;8(1):47–55. doi: 10.1128/EC.00276-08 19011120 10.1128/EC.00276-08 PMC2620750

[51] Ellis JE, Yarlett N, Cole D, Humphreys MJ, Lloyd D. Antioxidant defences in the microaerophilic protozoan Trichomonas vaginalis: comparison of metronidazole-resistant and sensitive strains. Microbiology. 1994;140 (Pt 9):2489–94. doi: 10.1099/13500872-140-9-2489 . 7952198 10.1099/13500872-140-9-2489

[52] Wassef R, Haenold R, Hansel A, Brot N, Heinemann SH, Hoshi T. Methionine sulfoxide reductase A and a dietary supplement S-methyl-L-cysteine prevent Parkinson’s-like symptoms. J Neurosci. 2007;27(47):12808–16. doi: 10.1523/JNEUROSCI.0322-07.2007 . 18032652 10.1523/JNEUROSCI.0322-07.2007 PMC6673285

[53] Liu CL, Hsia TC, Yin MC. s-Methyl cysteine enhanced survival of nerve growth factor differentiated PC12 cells under hypoxic conditions. Food & function. 2014;5(6):1125–33. doi: 10.1039/c3fo60689a . 24710107 10.1039/c3fo60689a

[54] Higuchi O, Tateshita K, Nishimura H. Antioxidative activity of sulfur-containing compounds in Allium species for human low-density lipoprotein (LDL) oxidation in vitro. Journal of agricultural and food chemistry. 2003;51(24):7208–14. doi: 10.1021/jf034294u . 14611195 10.1021/jf034294u

[55] Hsia TC, Yin MC. Post-Intake of S-Ethyl Cysteine and S-Methyl Cysteine Improved LPS-Induced Acute Lung Injury in Mice. Nutrients. 2016;8(8). doi: 10.3390/nu8080507 27548215 10.3390/nu8080507 PMC4997420

[56] Coombs GH, Westrop GD, Suchan P, Puzova G, Hirt RP, Embley TM, et al The amitochondriate eukaryote Trichomonas vaginalis contains a divergent thioredoxin-linked peroxiredoxin antioxidant system. The Journal of biological chemistry. 2004;279(7):5249–56. doi: 10.1074/jbc.M304359200 . 14630923 10.1074/jbc.M304359200

[57] McMillan PJ, Patzewitz EM, Young SE, Westrop GD, Coombs GH, Engman L, et al Differential inhibition of high and low Mr thioredoxin reductases of parasites by organotelluriums supports the concept that low Mr thioredoxin reductases are good drug targets. Parasitology. 2009;136(1):27–33. doi: 10.1017/S0031182008005131 . 18980703 10.1017/S0031182008005131

[58] Iulek J, Alphey MS, Westrop GD, Coombs GH, Hunter WN. High-resolution structure of recombinant Trichomonas vaginalis thioredoxin. Acta Crystallogr D Biol Crystallogr. 2006;62(Pt 2):216–20. doi: 10.1107/S0907444905039946 . 16421453 10.1107/S0907444905039946

[59] Reguera RM, Redondo CM, Perez-Pertejo Y, Balana-Fouce R. S-Adenosylmethionine in protozoan parasites: functions, synthesis and regulation. Molecular and biochemical parasitology. 2007;152(1):1–10. doi: 10.1016/j.molbiopara.2006.11.013 . 17196271 10.1016/j.molbiopara.2006.11.013

[60] Tehlivets O, Malanovic N, Visram M, Pavkov-Keller T, Keller W. S-adenosyl-L-homocysteine hydrolase and methylation disorders: yeast as a model system. Biochimica et biophysica acta. 2013;1832(1):204–15. doi: 10.1016/j.bbadis.2012.09.007 23017368 10.1016/j.bbadis.2012.09.007 PMC3787734

[61] Choumenkovitch SF, Selhub J, Bagley PJ, Maeda N, Nadeau MR, Smith DE, et al In the cystathionine beta-synthase knockout mouse, elevations in total plasma homocysteine increase tissue S-adenosylhomocysteine, but responses of S-adenosylmethionine and DNA methylation are tissue specific. J Nutr. 2002;132(8):2157–60. . 12163655 10.1093/jn/132.8.2157

[62] Caudill MA, Wang JC, Melnyk S, Pogribny IP, Jernigan S, Collins MD, et al Intracellular S-adenosylhomocysteine concentrations predict global DNA hypomethylation in tissues of methyl-deficient cystathionine beta-synthase heterozygous mice. J Nutr. 2001;131(11):2811–8. . 11694601 10.1093/jn/131.11.2811

[63] Lee HO, Wang L, Kuo YM, Gupta S, Slifker MJ, Li YS, et al Lack of global epigenetic methylation defects in CBS deficient mice. J Inherit Metab Dis. 2017;40(1):113–20. doi: 10.1007/s10545-016-9958-5 27444757 10.1007/s10545-016-9958-5 PMC5300059

[64] Thong KW, Coombs GH, Sanderson BE. S-adenosylmethionine and transmethylation reactions in trichomonads. Parasitol Res. 1987;73(3):193–8. . 3108875 10.1007/BF00578503

[65] Flint DH, Tuminello JF, Miller TJ. Studies on the synthesis of the Fe-S cluster of dihydroxy-acid dehydratase in escherichia coli crude extract. Isolation of O-acetylserine sulfhydrylases A and B and beta-cystathionase based on their ability to mobilize sulfur from cysteine and to participate in Fe-S cluster synthesis. J Biol Chem. 1996;271(27):16053–67. . 8663055 10.1074/jbc.271.27.16053

[66] Sato D, Nozaki T. Methionine gamma-lyase: the unique reaction mechanism, physiological roles, and therapeutic applications against infectious diseases and cancers. IUBMB Life. 2009; 61(11);1019–28. doi: 10.1002/iub.255 . 19859976 10.1002/iub.255

[67] Sato D, Yamagata W, Harada S, Nozaki T. Kinetic characterization of methionine gamma-lyases from the enteric protozoan parasite Entamoeba histolytica against physiological substrates and trifluoromethionine, a promising lead compound against amoebiasis. FEBS J. 2008;275(3):548–60. doi: 10.1111/j.1742-4658.2007.06221.x 18199285 10.1111/j.1742-4658.2007.06221.x

[68] Goyer A, Collakova E, Shachar-Hill Y, Hanson AD. Functional characterization of a methionine gamma-lyase in Arabidopsis and its implication in an alternative to the reverse trans-sulfuration pathway. Plant Cell Physiol. 2007;48(2):232–42. doi: 10.1093/pcp/pcl055 . 17169919 10.1093/pcp/pcl055

[69] Jakubowicz M. Structure, catalytic activity and evolutionary relationships of 1-aminocyclopropane-1-carboxylate synthase, the key enzyme of ethylene synthesis in higher plants. Acta Biochim Pol. 2002;49(3):757–74. . 12422245

[70] Berger LC, Wilson J, Wood P, Berger BJ. Methionine regeneration and aspartate aminotransferase in parasitic protozoa. Journal of bacteriology. 2001;183(15):4421–34. doi: 10.1128/JB.183.15.4421-4434.2001 11443076 10.1128/JB.183.15.4421-4434.2001 PMC95336

[71] Lowe PN, Rowe AF. Aminotransferase activities in Trichomonas vaginalis. Molecular and biochemical parasitology. 1986;21(1):65–74. . 3095639 10.1016/0166-6851(86)90080-0

[72] Lowe PN, Rowe AF. Aspartate: 2-oxoglutarate aminotransferase from trichomonas vaginalis. Identity of aspartate aminotransferase and aromatic amino acid aminotransferase. The Biochemical journal. 1985;232(3):689–95. 3879173 10.1042/bj2320689 PMC1152940

[73] Lloyd D, Coombs G.H. The catabolism of amino acids by Trichomonas vaginalis In: Lloyd D, Coombs G.H. and Paget T.A., editor. The Biochemistry and Molecular Biology of ‘Anaerobic’ Protozoa. Switzerland: Harwood Academic Publishers GmbH; 1989 p. 93–111.

[74] Wu G, Fiser A, ter Kuile B, Sali A, Muller M. Convergent evolution of Trichomonas vaginalis lactate dehydrogenase from malate dehydrogenase. Proceedings of the National Academy of Sciences of the United States of America. 1999;96(11):6285–90. 10339579 10.1073/pnas.96.11.6285 PMC26873

[75] Sakko M, Tjaderhane L, Sorsa T, Hietala P, Jarvinen A, Bowyer P, et al 2-Hydroxyisocaproic acid (HICA): a new potential topical antibacterial agent. International journal of antimicrobial agents. 2012;39(6):539–40. doi: 10.1016/j.ijantimicag.2012.02.006 . 22483561 10.1016/j.ijantimicag.2012.02.006

[76] Sakko M, Moore C, Novak-Frazer L, Rautemaa V, Sorsa T, Hietala P, et al 2-hydroxyisocaproic acid is fungicidal for Candida and Aspergillus species. Mycoses. 2014;57(4):214–21. doi: 10.1111/myc.12145 . 24125484 10.1111/myc.12145

[77] Sakko M, Tjaderhane L, Sorsa T, Hietala P, Rautemaa R. 2-hydroxyisocaproic acid (HICA) is bactericidal in human dental root canals ex vivo. Int Endod J. 2016 doi: 10.1111/iej.12639 . 27004733 10.1111/iej.12639

[78] Nieminen MT, Hernandez M, Novak-Frazer L, Kuula H, Ramage G, Bowyer P, et al DL-2-hydroxyisocaproic acid attenuates inflammatory responses in a murine Candida albicans biofilm model. Clinical and vaccine immunology: CVI. 2014;21(9):1240–5. doi: 10.1128/CVI.00339-14 24990903 10.1128/CVI.00339-14 PMC4178561

[79] Lang CH, Pruznak A, Navaratnarajah M, Rankine KA, Deiter G, Magne H, et al Chronic alpha-hydroxyisocaproic acid treatment improves muscle recovery after immobilization-induced atrophy. Am J Physiol Endocrinol Metab. 2013;305(3):E416–28. doi: 10.1152/ajpendo.00618.2012 . 23757407 10.1152/ajpendo.00618.2012

[80] Morada M, Smid O, Hampl V, Sutak R, Lam B, Rappelli P, et al Hydrogenosome-localization of arginine deiminase in Trichomonas vaginalis. Mol Biochem Parasitol. 2011;176(1):51–4. doi: 10.1016/j.molbiopara.2010.10.004 21074581 10.1016/j.molbiopara.2010.10.004 PMC3026898

[81] Fyfe PK, Westrop GD, Ramos T, Muller S, Coombs GH, Hunter WN. Structure of Leishmania major cysteine synthase. Acta Crystallogr Sect F Struct Biol Cryst Commun. 2012;68(Pt 7):738–43. doi: 10.1107/S1744309112019124 22750854 10.1107/S1744309112019124 PMC3388911

