# Supplementary figures and images for: Metabolomic profiling and stable isotope labelling of Trichomonas vaginalis and Tritrichomonas foetus reveal major differences in amino acid metabolism including the production of 2-hydroxyisocaproic acid, cystathionine and S-methylcysteine
Source: PLoS One. 2017 Dec 21;12(12):e0189072. doi: 10.1371/journal.pone.0189072 (PMC5739422; doi:10.1371/journal.pone.0189072)

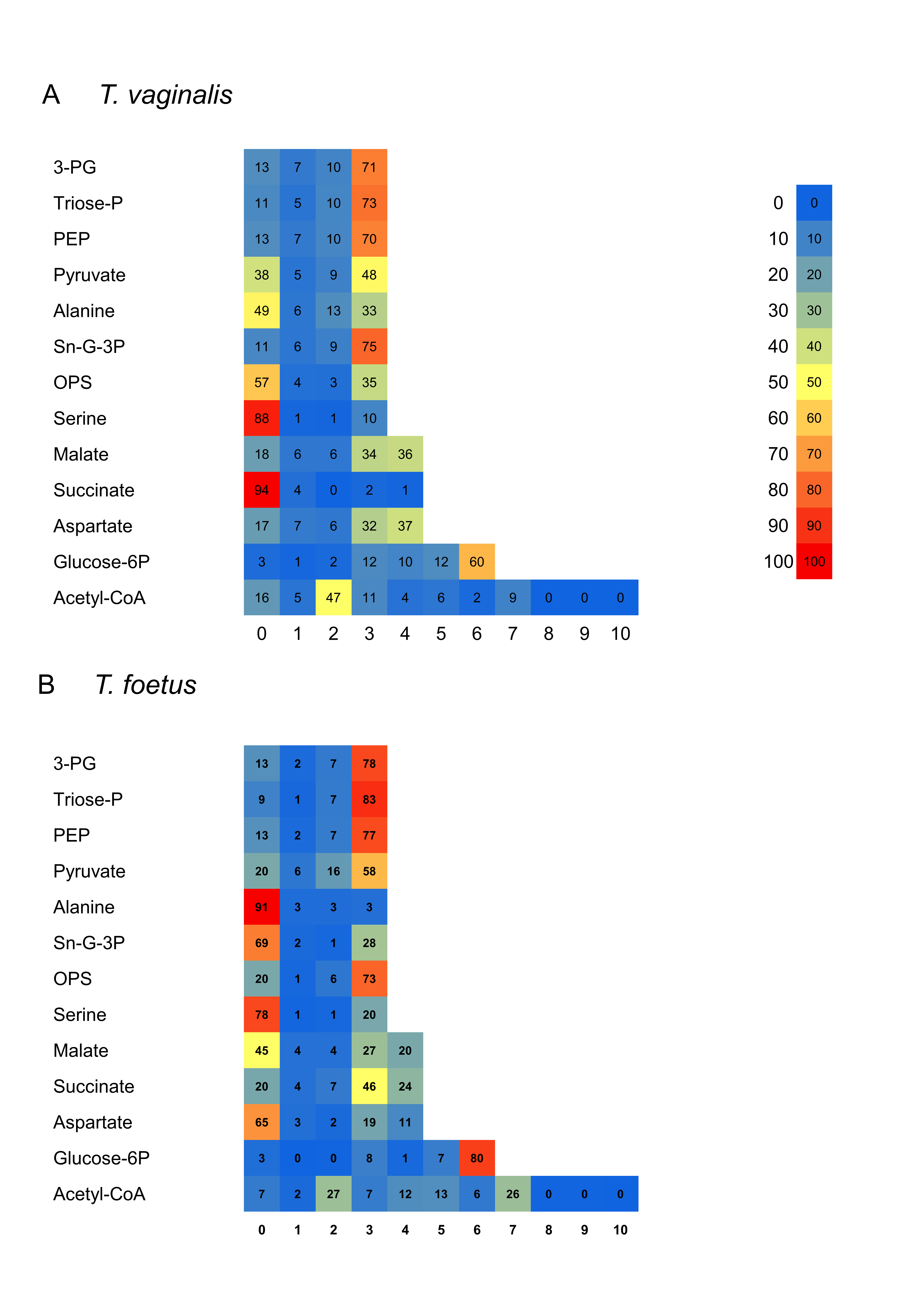

Supplement: S1 Fig — T. vaginalis and T. foetus were grown for 20 h at 37°C in MDM containing 100% D-[U-13C6] glucose as the only carbohydrate carbon source and cell extracts were analysed by LCMS. Heat maps of the percentage of unlabelled metabolites and isotopologues with increasing number of 13C atoms are shown for (A) T. vaginalis and (B) T. foetus. Numbers on the heat map refer to percentage labelling values. (TIF) [file pone.0189072.s007.TIF]

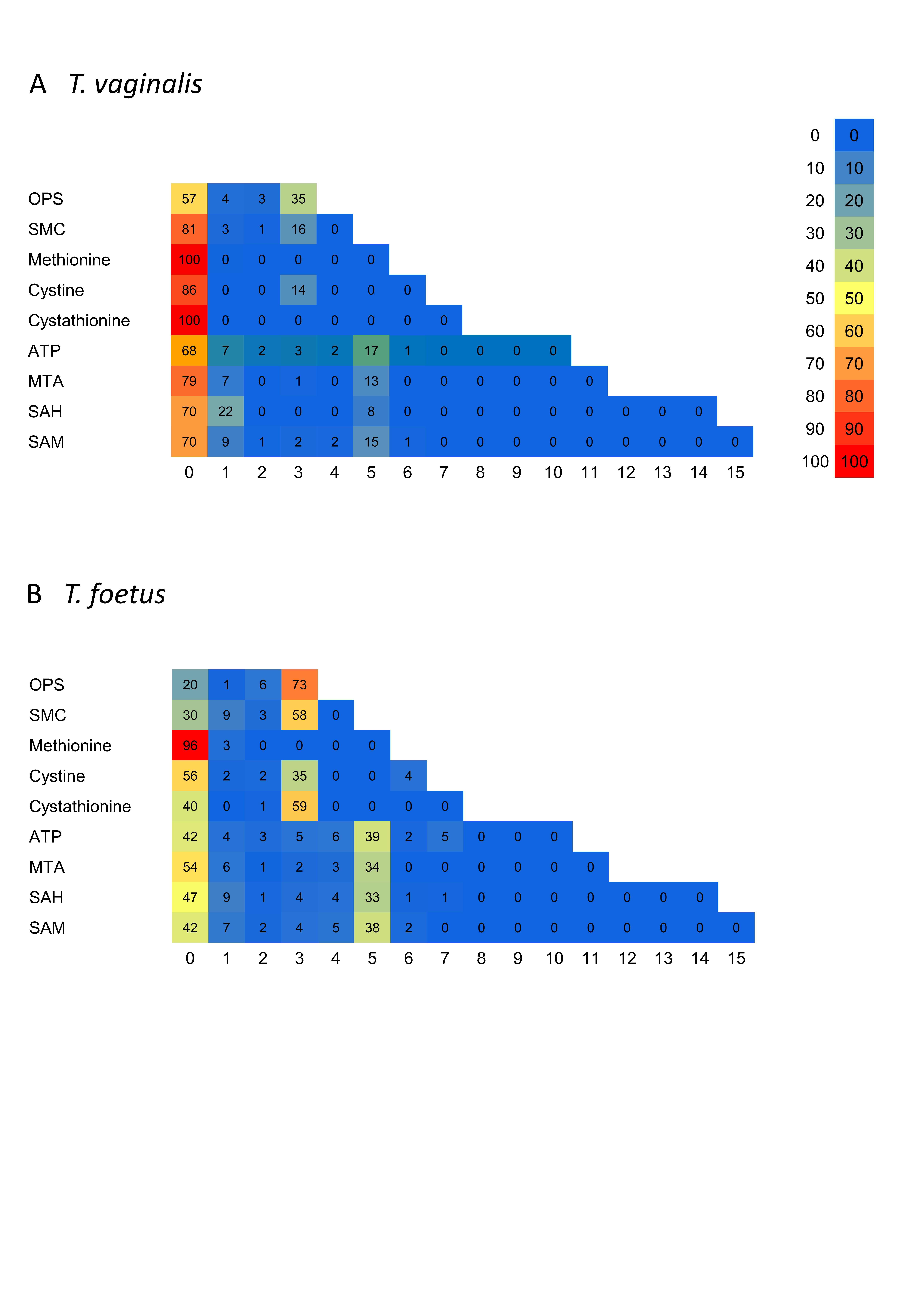

Supplement: S2 Fig — Heat maps of the percentage of unlabelled metabolites and isotopologues with increasing number of 13C atoms are shown for (A) T. vaginalis and (B) T. foetus. (TIF) [file pone.0189072.s008.TIF]

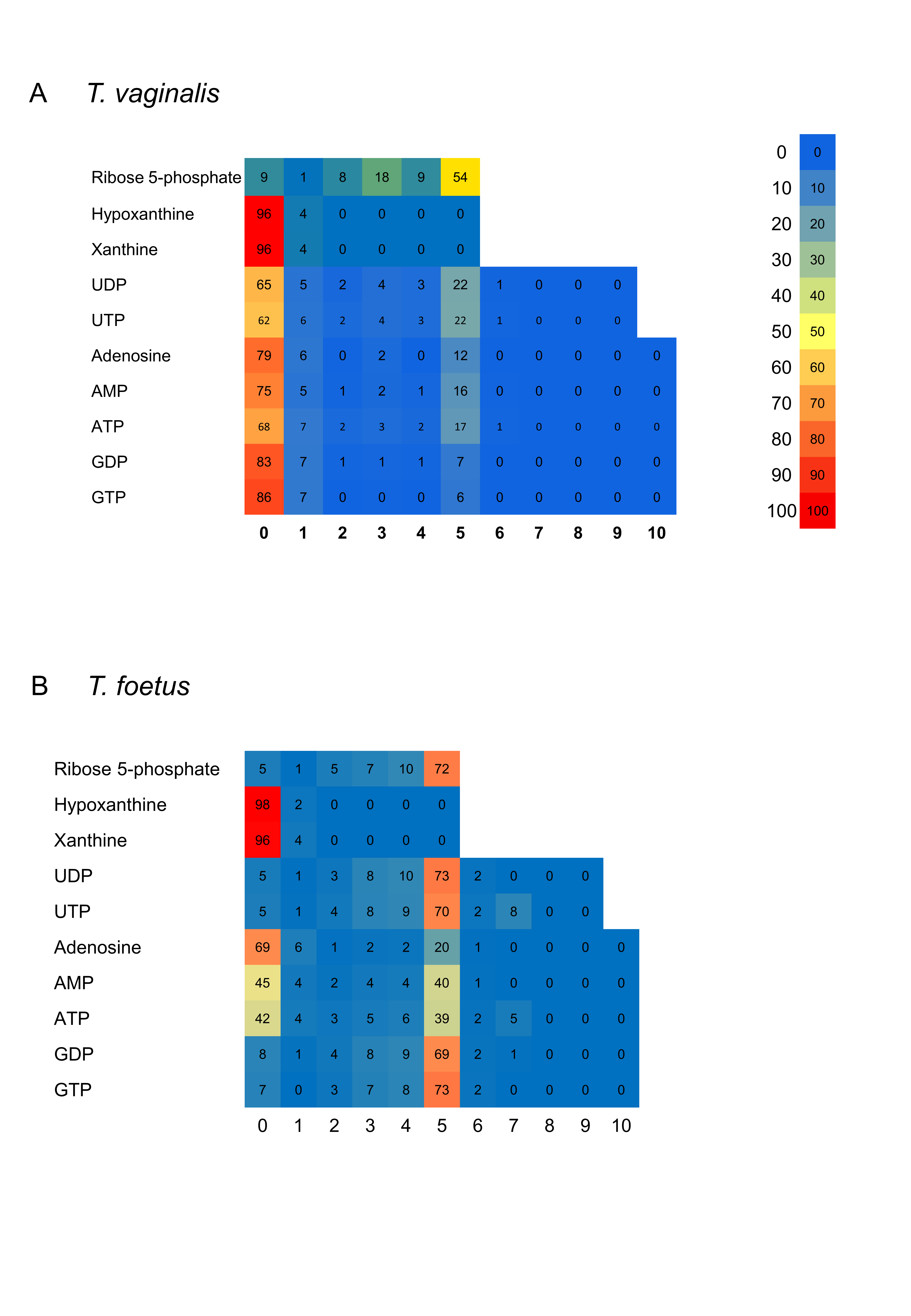

Supplement: S3 Fig — Heat maps of the percentage of unlabelled metabolites and their isotopologues with increasing numbers of 13C atoms are shown for (A) T. vaginalis and (B) T. foetus. (TIF) [file pone.0189072.s009.TIF]

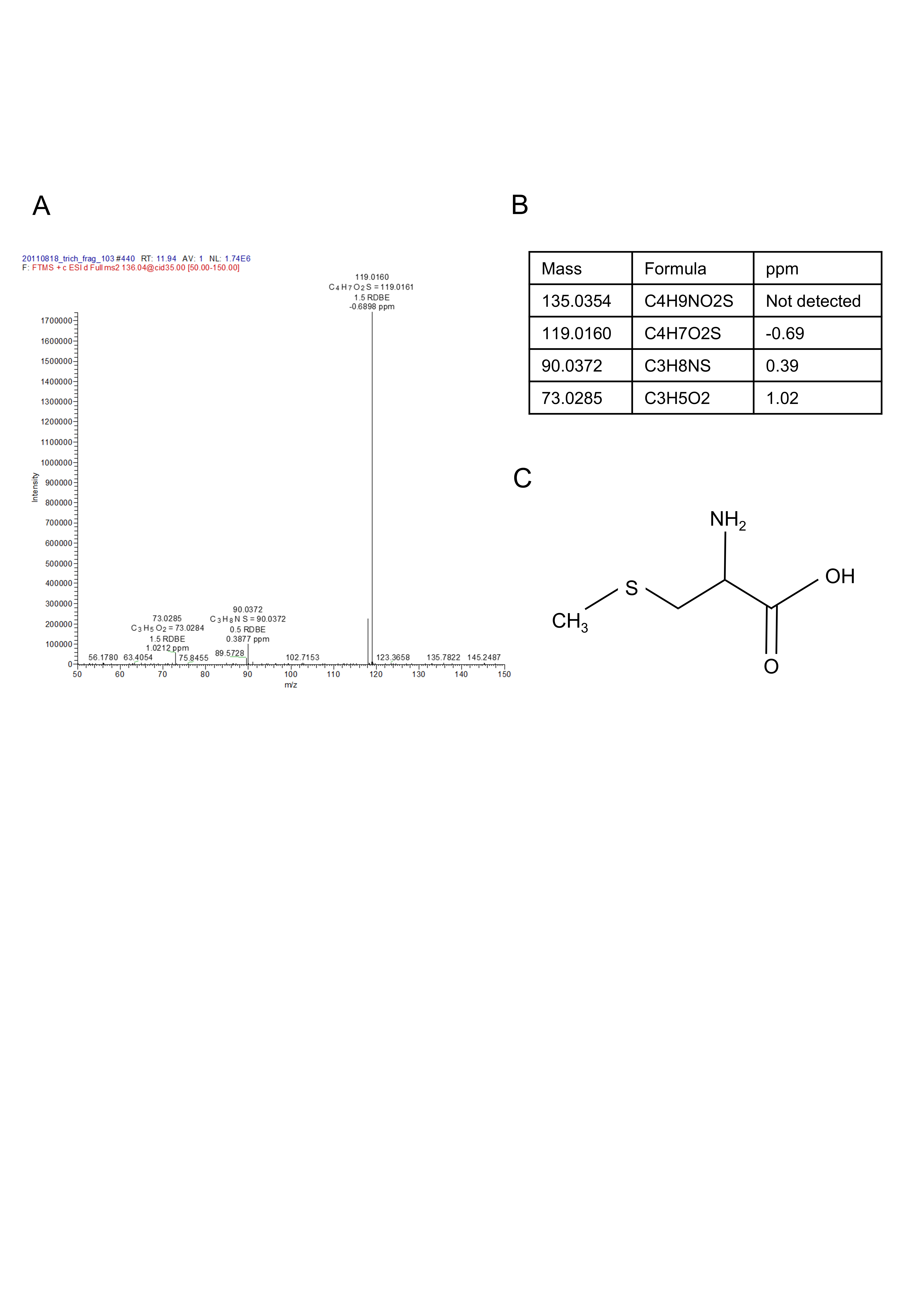

Supplement: S4 Fig — A metabolite with predicted formula C4H9NO2S was analysed by MS2. The full length molecule was not seen in the mass spectrum. Fragments detected and their predicted formula are shown (A) in the mass spectrum and (B) in the table. The data is consistent with the structure of S-methyl-cysteine (C). (TIF) [file pone.0189072.s010.TIF]

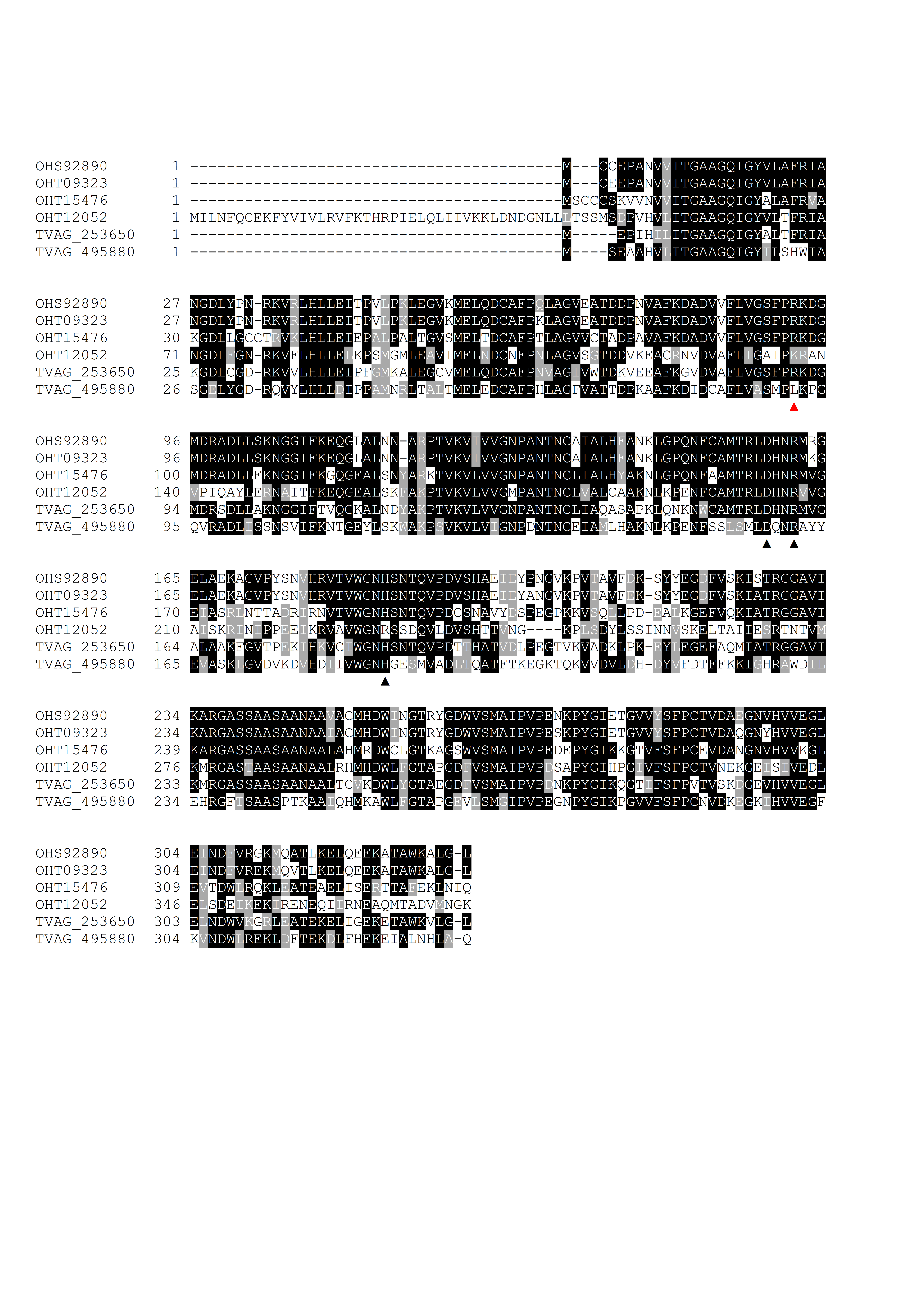

Supplement: S5 Fig — Four putative isoforms of T. foetus MDH are aligned with MDH (TVAG_253650) and LDH (TVAG_49988). Conserved residues are in white type on a black background. Conservative amino acid differences are in white type on a grey background. The red arrow below the line indicates a residue found to be a major determinant of substrate specificity, this is arginine in T. vaginalis MDH but leucine in T. vaginalis LDH. All isoforms found in T. foetus have a positively charged residues at this position and are therefore predicted to have MDH activity. Black arrows indicate conserved residues involved in substrate binding or enzyme activity. (TIF) [file pone.0189072.s011.tif]

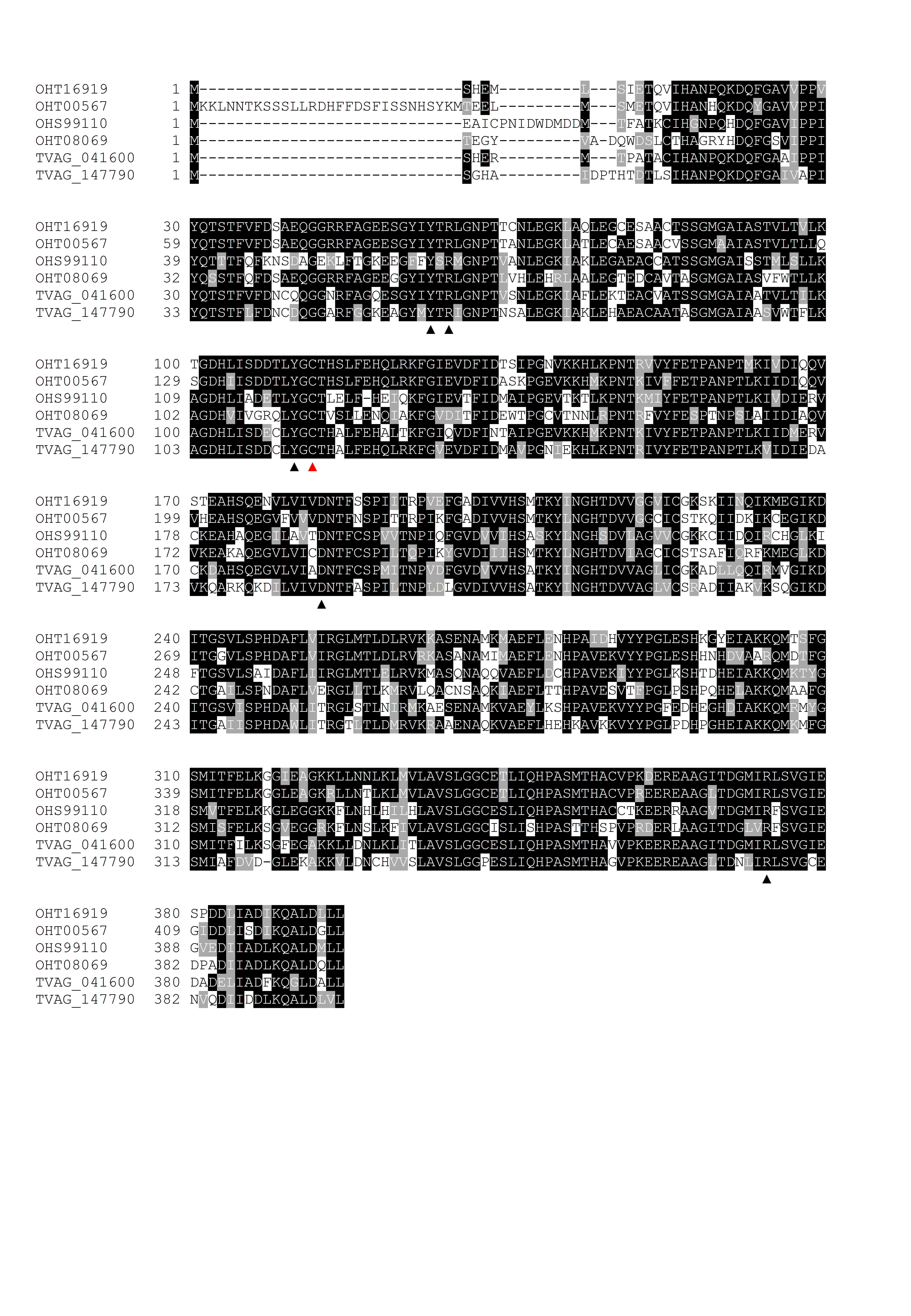

Supplement: S6 Fig — Sequences obtained by tblastn search of the T. foetus and T. vaginalis genome sequences with the T. vaginalis MGL1 (TVAG_041600) as a query sequence. Conserved residues are in white type on a black background. Conservative amino acid differences are in white type on a grey background. Symbols: ▲, active site residues involved in PLP binding; ▲, cysteine residue implicated in methionine binding. (TIF) [file pone.0189072.s012.tif]
